# Shear and Breathing Modes of Layered Materials

**DOI:** 10.1021/acsnano.0c10672

**Published:** 2021-08-09

**Authors:** Giovanni Pizzi, Silvia Milana, Andrea C. Ferrari, Nicola Marzari, Marco Gibertini

**Affiliations:** † Theory and Simulation of Materials (THEOS), and National Centre for Computational Design and Discovery of Novel Materials (MARVEL), École Polytechnique Fédérale de Lausanne, CH-1015 Lausanne, Switzerland; ‡ Cambridge Graphene Centre, University of Cambridge, Cambridge CB3 OFA, U.K.; ∥ Dipartimento di Scienze Fisiche, Informatiche e Matematiche, University of Modena and Reggio Emilia, IT-41125 Modena, Italy; § Department of Quantum Matter Physics, University of Geneva, CH-1211 Genéve, Switzerland

**Keywords:** layered materials, Raman, infrared, multilayer, fan diagrams, spectroscopy, fingerprint, space groups

## Abstract

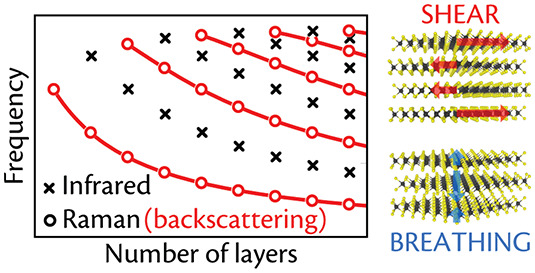

Layered materials (LMs), such as graphite, hexagonal boron nitride, and transition-metal dichalcogenides, are at the center of an ever-increasing research effort, due to their scientific and technological relevance. Raman and infrared spectroscopies are accurate, non-destructive approaches to determine a wide range of properties, including the number of layers, *N*, and the strength of the interlayer interactions. We present a general approach to predict the complete spectroscopic fan diagrams, *i.e.*, the relations between frequencies and *N* for the optically active shear and layer-breathing modes of any multilayer comprising *N* ≥ 2 identical layers. In order to achieve this, we combine a description of the normal modes in terms of a one-dimensional mechanical model, with symmetry arguments that describe the evolution of the point group as a function of *N*. Group theory is then used to identify which modes are Raman- and/or infrared-active, and to provide diagrams of the optically active modes for any stack composed of identical layers. We implement the method and algorithms in an open-source tool to assist researchers in the prediction and interpretation of such diagrams. Our work will underpin future efforts on Raman and infrared characterization of known, and yet not investigated, LMs.

Layered materials (LMs) are at the center of an ever-growing research effort due to the variety of their potential applications in a wide range of fields.^1^ There are at least 5000 materials that are layered,^2^ with at least 1800 that are exfoliable,^2−5^ and even more that could be synthesized.^6−9^ However, only a very small fraction of these have been experimentally investigated to date, such as graphene, hexagonal boron nitride (hBN), black phosphorus (BP), transition metal dichalcogenides (TMDs), InSe and other monochalcogenides, MAXenes, and very few others. When a given bulk LM (B-LM) is exfoliated into a multilayer (ML), the optical and electronic properties change with the number of layers (*N*). For a given *N*, the properties can be tuned by varying the relative orientation of the layers.^10−13^ For a given *N* and orientation, properties can also be changed by arranging different LMs in heterostructures (LMHs).^14−19^ The degrees of freedom are such that it will take decades, if ever, before all possible LMs will be exfoliated, and investigated when arranged in LMHs, as a function of *N* and of relative orientation. Due to the extraordinary range of properties that can be addressed, it is essential to develop approaches to identify *N* in any given assembly or device.

Techniques to measure *N* based on optical contrast^20^ have been developed. However, they depend on the substrate and do not readily provide information such as strain or doping. A more informative approach is offered by Raman^21^ and infrared (IR)^22^ spectroscopies that probe phonons.

In particular, in LMs there are two fundamentally different sets of modes: Those coming from the relative motion of the constituent atoms within each layer, usually found at high frequencies (>100 cm^–1^),^21^ and those due to relative motions of the layers themselves, either perpendicular, C (or shear) modes, or parallel, layer-breathing (LB) modes (LBMs), to their normal.^21,23−25^ Several studies have identified these modes in a limited set of ML-LMs, such as ML-graphene,^26−29^ TMDs^30^ (*e.g.*, MoS_2_,^31,32^ MoSe_2_,^33^ WS_2_,^34^ WSe_2_,^35^ MoTe_2_,^36,37^ ReS_2_,^38−40^ ReSe_2_,^40,41^ PtS_2_^42^), NbSe_2_,^43−45^ hBN,^46^ phosphorene,^47−49^ Bi_2_X_3_,^50^ and metal chalcogenides (*e.g.*, GaSe,^51,52^ InSe,^52^ and SnS_2_^53^).

The optically active (Raman or IR) modes can be plotted as a function of *N*, in a graph that looks like a fan, thus called fan diagram.^26^ The experimental data can be explained with a linear chain model,^26,54^ whereby each plane is linked to the next by a spring, modeled by scalar interlayer force constants corresponding to a motion parallel (C) or perpendicular (LB) to the planes.^26^

Here, we extend the linear chain model to every possible exfoliable LM composed of identical layers by implementing a group-theory approach. We start from the B-LM symmetry properties to derive a general tensorial expression for the interlayer force constants. We show how to derive the evolution of the point group for any *N*, knowing the space group of the B-LM, considered as the repetition of a single layer (1L), stacked recursively. This is then used to assign each normal mode to a given irreducible representation of the corresponding point group, in order to assess its optical activity and obtain the fan diagram of each LM. Finally, we provide an online tool, available on Materials Cloud^55^ at the address https://materialscloud.org/work/tools/layer-raman-ir, that accepts user-supplied structures and computes on the fly the corresponding fan diagram and symmetry-compliant form of the interlayer force constants. Our work provides the interpretation of the C and LBM patterns measured in any LM composed of identical layers, either already experimentally investigated, or, more importantly, any of those that will be studied in the future.

## 1 Fan Diagrams: Prediction and Interpretation

A fan diagram is a plot of the normal-mode frequencies associated with the rigid relative motion of the layers in an ML-LM, as a function of *N*. The fan diagram frequencies are a fingerprint of each material. Their trend as a function of *N* depends on the atomic structure and the symmetry, both of the ML-LM system and of the corresponding B-LM.

We develop a theoretical model to interpret the experimental results and to assess the origin and character of these vibrational modes and their expected optical activity. Such a model needs a number of components:

1. We need an approach to compute the normal vibrational modes of ML-LMs and their frequencies, using a model that can capture the system geometry and only depends on a few material parameters, such as the force constants between each pair of layers.
2. We need to identify and extract the layers of ML-LMs from the B-LM structure and analyze their crystal symmetry. Given the space group of B-LMs, we need to determine all possible symmetries of ML-LM system with a given *N*.
3. We need to exploit the symmetry information to identify the optical activity of each normal mode (*i.e.*, if the mode is Raman- or IR-active, and, if so, if it can be detected in the most commonly used back-scattering geometry^21,56^). We use group theory to classify each mode, assigning it to the irreducible representation to which it belongs, thus determining its optical activity.
4. We then combine points 1–3 above in a single model to enable the interpretation of the experimental data.

### 1.1 Definition of a Layered Material and Nomenclature

We are interested in modeling the vibrational properties of LMs when layers move as rigid units as a consequence of the strong covalent bonds between atoms in a given layer, as opposed to the weak van der Waals interactions keeping layers together.

In this limit, C and LB vibrations can be described in terms of interlayer force constants, acting as restoring forces between nearby layers.

In order to limit the number of parameters in the model and to make use of crystal symmetry and space-group concepts to predict the normal modes and their optical activity, we consider LMs with a sufficiently regular stacking (to be described below, in particular focusing on LMs composed of identical layers), which covers the majority of naturally occurring LMs.

Here, we cover MLs comprising *N* ≥ 2 identical layers.

In refs (29, 57, 58), linear chain models were applied to twisted graphene MLs and graphene-MoS_2_ or hBN-WS_2_ stacks. We note that these approaches are specific to the systems considered. Our model could be numerically extended to any LM and LMH. Group theory can still be used to obtain the form of the interlayer mechanical couplings that enter the equations of motion.^59,60^ These can then be solved numerically, to finally assign the infrared or Raman character of the modes using symmetry arguments. Stacking in LMHs lowers the symmetry, lifting most symmetry constraints on the optical activity of modes. Group theory alone could predict modes to be active even if the corresponding intensity might be negligible. Thus, further computation of the optical-coupling matrix elements becomes essential. In non-recursive stacking sequences, especially when involving different layers, more parameters enter the description of interlayer force constants (with a different force-constant matrix for each layer pair and for each possible relative orientation of the two), which can be extracted from additional first-principles simulations, in order to reduce the number of free parameters in the model.

We follow a practical approach, giving a brief explanation of the important symmetry properties of LMs. Ref (61) reported a complete treatment with formal definitions and proofs. Because the nomenclature used in the experimental literature of ML fan diagrams often differs from that used in the crystallographic community,^61^ we also provide a mapping between the names used in the two communities, where appropriate.

The International Union of Crystallography calls ML-LMs “polytypes” (see ref (62) for a formal definition). A theory to describe these ML-LMs, based solely on the symmetry of each layer and on the symmetry relation between subsequent layers, was developed in refs (63 and 64).

Here, we limit our study to LMs where all layers are identical and can be mapped onto each other through coincidence operations, defined as isometries (*i.e.*, space transformations that preserve the distance between any two points) bringing a layer of the ML-LM onto the next one. As already noted above, this excludes, *e.g.*, B-LMHs formed by different LMs, as in the case of franckeite,^65,66^ but is the typical case for exfoliable materials.

**Figure 1:**
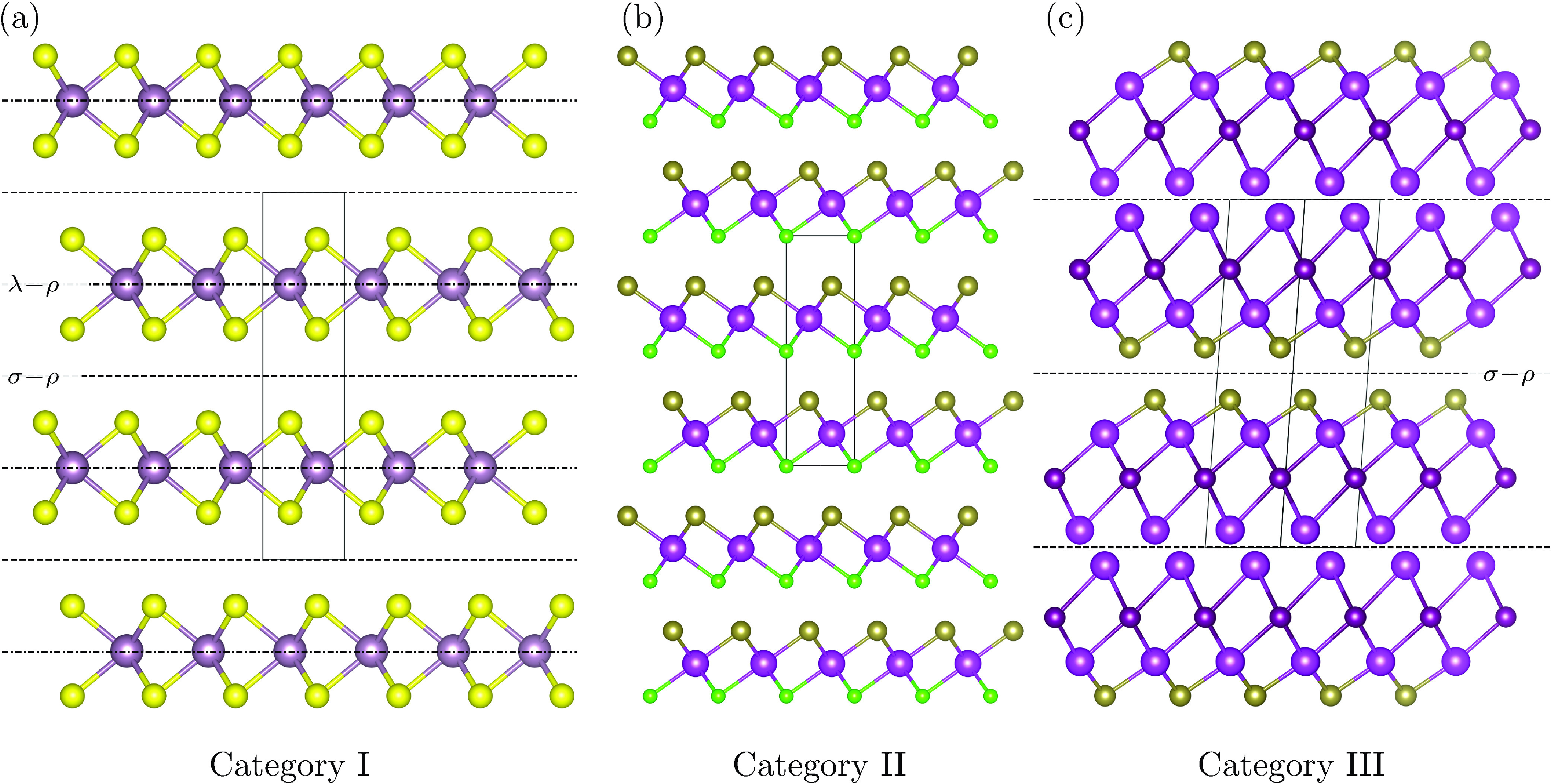
3 LM categories allowed from a symmetry point of view, for MDO polytypes of equivalent layers. (a) Category I: each layer is non-polar along the stacking direction (*i.e.*, it has a symmetry operation that flips it upside down), such as in MoS_2_ (structure from the Crystallography Open Database (COD^67^), code 9007660). Mo atoms are shown in violet and S atoms in yellow. (b) Category II: each layer is polar along the stacking direction, and all layers are oriented in the same direction, such as in BiTeCl (structure from the Inorganic Crystal Structure Database (ICSD^68^), code 79362). Bi atoms are shown in light purple, Te atoms in brown, and Cl atoms in green. (c) Category III: each layer is polar along the stacking direction, and they stack in alternating polarization directions, such as Bi_2_TeI (ICSD^68^ code 153858). Bi atoms are shown in light purple, Te atoms in brown, and I atoms in dark purple. Symmetry planes for layer-order-changing operations (ρ planes) are indicated with dashed lines (σ operations) or dotted-dashed lines (λ operations). Note that the LM in Category III is for illustrative purposes: depending on the nature of the chemical bonding, this could be considered to be 3 non-equivalent layers (2 BiTeI layers analogous to those of Category II, and 1 layer of Bi atoms).

The coincidence operation that brings one layer onto the next might not be the same for all layers (*e.g.*, if the first layer is mapped onto the second one by a translation, while the second is mapped onto the third by a rotation). Again, with the goal being to limit the number of parameters in the model, we then consider an additional requirement by limiting our analysis to maximum degree of order (MDO) polytypes. These are LMs where the coincidence operation is total; *i.e.*, it is the same between any pair of adjacent layers. As a consequence,^61^ in an MDO polytype any triplet of subsequent layers is equivalent, whereas it is not true that every pair is equivalent, as shown in the example of Figure 1c for Bi_2_TeI. Because any triplet is equivalent, MDO polytypes have only one or two independent interatomic force-constant tensors that occur between nearby layers in the triplet, while all other tensors can be reconstructed using symmetry arguments. If the coincidence operation is not total, the relative arrangement of atoms in pairs of subsequent layers could be different, leading to different interactions between them, even if this almost never occurs in naturally occurring exfoliable materials.

Within these constraints, we can classify all LMs in three categories,^61^ shown with three examples in Figure 1. Table 1 provides a summary of the type of operations in each of the categories of Figure 1.

**Table 1:**
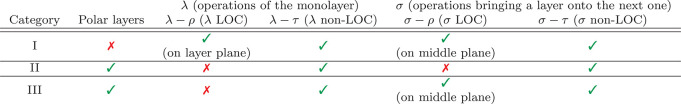
Summary Table of the Type of Operations in Each of the Categories of Figure 1

We first consider the case where each layer is non-polar along the stacking direction (*i.e.*, it has a symmetry that flips it upside down) and then when it is polar. In the non-polar case, only one possibility exists (Category I, Figure 1a). In the polar case, there are two options: either the polarity has the same orientation for all layers (Category II, Figure 1b) or it alternates between layers (Category III, Figure 1c). These three categories have very different sets of symmetry operations. We note that Category III, while considered here for completeness from a symmetry point of view, never occurs to the best of our knowledge, for the most common LMs, such as graphite, hBN or TMDs.

We define the planes of the layers in the LM as the “horizontal” direction, and the stacking direction of the LM as the “vertical” or *z* direction (note that the third vector of the bulk unit cell might not be orthogonal to the plane of the layers, see, *e.g.*, Figure 1c).

We then distinguish the symmetry operations of 1L-LMs (called λ symmetries in ref (61)) and the coincidence operations bringing a layer onto the next one (σ symmetries).

Any symmetry operation can either change the sign of any vertical coordinate, *i.e.*, flip the layer upside-down (called ρ operations,^61^ like inversion, roto-reflections, reflections under horizontal planes, or two-fold rotations with an horizontal axis), or not change the sign of the vertical-direction coordinates (called τ operations,^61^*e.g.*, translations, rotations with a vertical axis, or reflections under vertical planes; these form a subgroup). Because all ρ operations change the stacking order of the layers in a LM (*e.g.*, a stacking 1-2-3-1-2-3 becomes 3-2-1-3-2-1), in the following we call them layer-order-changing (LOC) operations,^69^ whereas we call the τ ones non-LOC operations.

With these definitions, for non-polar layers (Figure 1a), both λ and σ operations can be either LOC or non-LOC.^61^ Thus, we can formally define the vertical *z* coordinate of each layer as that of its inversion center (or reflection plane or rotation axis). The plane with this *z* coordinate is called the layer plane.^61^ Then, LOC operations can either be λ, and, in this case, their symmetry elements are on a layer plane, or σ, and they must lie on planes halfway between layer planes, as shown in Figure 1a. Henceforth, we will call these planes “middle planes”. Non-LOC σ operations (bringing one layer onto the next) are always combined with a translation along the vertical direction.

Polar layers do not have any symmetry operation that flips the *z* coordinates, so all λ operations are non-LOC. However, whereas in Category II of Figure 1 all σ coincidence operations are non-LOC (because the polarity direction is never reversed), in Category III all σ coincidence operations must be LOC (changing polarization orientation between consecutive layers) and they also lie on middle planes. In Category II and III we cannot univocally define a layer plane because there is no layer inversion plane. However, by symmetry, it is possible to define two sets of middle planes in Category III.

This distinction of three categories is thus important for modeling the interlayer force constants. In Categories I and II all pairs of layers are equivalent. Therefore, the same interlayer force constant matrix (up to a similarity transformation) can be used to describe the interaction between any pair of nearby layers. In Category III there are two different interlayer force constant matrices, depending on whether the polarizations of the two neighboring layers are pointing inwards or outwards with respect to the van der Waals gap between them. Although this classification is extremely important to simplify the description of C and LB modes by distinguishing possible situations concerning the coincidence operation bringing one layer into the next one, a full account of the symmetries of the ML, discussed in Section 1.2, is needed to predict possible mode degeneracies and their optical activity.

When a LM satisfies all the conditions above, the description of its vibrational properties and of the symmetries of the corresponding ML-LMs is greatly simplified and can be carried out analytically or semi-analytically. For this reason, henceforth we focus only on this class of LMs. Nonetheless, our approach can be extended to any LMs or LMHs, although a numerical treatment might be needed, with decreased predictivity due to the increased number of free parameters associated with more symmetry-inequivalent force-constant matrices. Under the assumption of a rigid motion of the layers, if we relax the conditions discussed above, the interlayer force constants between neighboring layers might be all different if several LMs are stacked together. In addition, the symmetry constraints are less effective in reducing the number of independent parameters entering such force constants, owing to the lower symmetry for arbitrary-angle stacking configurations.

### 1.2 How To Derive the Point Group of a Multilayer Layered Material

We now consider how to obtain the point group of a ML-LM with *N* layers, as needed to predict its optical activity, given the point group of its parent B-LM that extends periodically in the direction orthogonal to the layers.

We call *n*_c_ the number of layers in the B-LM conventional cell, and *n*_p_ that in the B-LM primitive cell. By definition, the primitive unit cell is the smallest unit cell that, when repeated periodically, covers the full space without voids or overlaps.^70^ It is not required for the axes of the cell to be along high-symmetry directions.^70^ The conventional cell is the smallest cell that also captures the symmetry of the system (*i.e.*, with lattice vectors along symmetry elements).^70^ In some cases, this leads to a larger unit cell than the primitive one, so that, in general, *n*_c_ ≥ *n*_p_.^70^ For Bernal-stacked graphite, the primitive and conventional cells coincide and they both contain two layers, *n*_c_ = *n*_p_ = 2. Rhombohedral graphite has a rhombohedral primitive cell with only one layer (*n*_p_ = 1), while the conventional hexagonal cell has three layers (*n*_c_ = 3).

We define the “stacking index” as an integer indexing the layers so that, *e.g.*, if a layer has stacking index *l*, then the next layer (in the positive vertical direction) has *l* + 1.

The stacking direction, orthogonal to the planes of the layers, is unique. Thus, for some crystal systems it is prescribed by symmetry. In particular, in tetragonal, hexagonal, and trigonal systems, it must be along the *n*-fold characteristic symmetry axis (*e.g.*, the *c* axis for tetragonal systems). If this was not the case, *n*-fold rotations (with *n* > 2) would bring the stacking direction into other distinct ones, which would violate its unicity. The same arguments imply that cubic systems are not compatible with a layered structure.^69,70^ If a given direction, say *z*, were the stacking one, then also *x* and *y* should be by symmetry, as in cubic systems all principal directions are equivalent. Therefore, we do not consider cubic systems henceforth.

In orthorhombic, monoclinic, and triclinic systems, the stacking direction is not prescribed by symmetry, therefore the space group alone is not sufficient to characterize them. Instead, we need to consider all inequivalent settings, *i.e.*, possible non-conventional choices for the origin and lattice vectors with respect to symmetry elements. We consider all settings that are typically discussed in crystallography,^71,72^ identified by their Hall number.^73^ This ranges from 1 to 488 if we exclude cubic systems. *E.g*., space group 17 (*P*222_1_, a primitive orthorhombic system with 1 screw axis and no mirror symmetry) can be realized in 3 different settings, depending on the direction of the screw axis, with Hall numbers 109, 110, 111 for the screw axis aligned along the third, first, and second cell axes, respectively. Here, we assume the stacking direction to be orthogonal to the first two lattice vectors. Then, for setting P222_1_ with Hall number 109, the 2_1_ screw axis is along the stacking direction, and an ML in this Hall setting has different symmetry properties than one with Hall numbers 110 and 111, corresponding to *P*2_1_22 and *P*22_1_2, which are equivalent for our purposes, because, in both cases, the screw axis is horizontal.

We now present a strategy to obtain “compatibility relations”, *i.e.*, rules determining the possible point groups *G*_*N*_ of an ML-LM as a function of *N*, by knowing the B-LM space group and setting (thus also the B-LM point group *G*_b_), and the direction along which the material is layered. This enables us to identify which point-group operations of the B-LM (*i.e.*, of *G*_b_) are part of *G*_*N*_.

For *N* ≥ *n*_c_, *G*_*N*_ is a subgroup of *G*_b_ because any operation of the ML-LM must also be one of the B-LM for an MDO polytype. For *N* < *n*_c_, this statement is not always true, as we later discuss, so this requires an independent treatment. *n*_c_ ranges from 1 to 3 for the most-studied LMs, such as 1-T TMDs like PtS_2_ (*n*_c_ = 1), or MoS_2_, hBN, and Bernal-stacked graphite (*n*_c_ = 2), or rhombohedral graphite (*n*_c_ = 3). Because the modes plotted in the fan diagrams (*i.e.*, relative rigid oscillations of the layers) only exist for *N* ≥ 2, the condition *N* ≥ *n*_c_ is, thus, not a strong limitation.

We first consider non-LOC operations. Non-LOC σ operations (σ–τ) can never be symmetries of a finite ML-LM because they map each layer with stacking index *l* onto that with *l* + 1. For λ non-LOC (λ–τ) symmetries, these non-LOC layer-invariant operations form a group^69^ that we call the layer-invariant point group, *G*_I_, which is a subgroup of *G*_b_. Because all elements of *G*_I_ leave each layer invariant individually (*i.e.*, they map each layer onto itself^69^), they are also symmetry operations of the ML for any *N*. Thus, *G*_I_ is a subgroup of *G*_*N*_. Given a B-LM space group, in order to obtain *G*_I_ we need to consider all B-LM symmetry operations that are non-LOC. For each of these, we take only their rotational part, and consider the point group that they form. *G*_I_ for all space groups and settings are reported in Table 3.

To obtain the complete *G*_*N*_, we have to complement *G*_I_ with all LOC (ρ) operations of the ML-LM, which are a subset of the LOC operations of the B-LM. For Category II, no LOC operations exist in B-LM, see Table 1. Therefore, there are no additional operations to consider and *G*_*N*_ = *G*_I_, independent of *N* and *n*_c_. For Categories I and III, LOC operations exist, and we need to select the B-LM LOC operations compatible with a finite ML-LM.

We focus on Category I because, as explained in Section 5.3, Category III can be considered as a special case of Category I for the determination of the point group. For an ML-LM with *N* layers, if an inversion center for a LOC operation exists, this must be the plane of the central layer if *N* is odd (*e.g.*, layer with stacking index 3 if *N* = 5), or the middle plane between the two central layer planes if *N* is even. Therefore, *G*_*N*_ will be obtained complementing *G*_I_ only with LOC operations that have inversion planes on a layer or middle plane.

For Category I, there is always at least one operation with such a plane. If *n*_c_ is odd, any LOC can be considered as having symmetry both on a layer plane or (with a different fractional translation) on a middle one, so all such LOCs can be included when computing *G*_*N*_. However, for even *n*_c_, LOCs can either have inversion on a layer, or on a middle plane. Depending on the parity of *N*, two point groups might alternate, corresponding to which set of LOCs is compatible with *N*.

Table 3 reports the complete set of possible *G*_*N*_ for each Hall setting and for *n*_c_ = 1, ..., 6. Table 3 often gives two symbols (/ or ×) instead of one or two possible *G*_*N*_. These symbols indicate cases in which it is impossible to create a ML in that setting with the specified *n*_c_ in the conventional cell. The meaning of the two symbols is explained in the Table caption and, in detail, in Sections 5.1, 5.2.

We now illustrate with a few examples how to use Table 3. We stress that the online tool presented in Section 3 performs the symmetry analysis automatically without the need to check Table 3.

Given a LM, we first need to identify its layers and determine in which category of Figure 1 it falls, depending on the 1L-LM symmetry.

Let us start with an example for Category I. If we consider MoS_2_ (in its 2H phase), hBN, or Bernal graphite, in all these cases the 1L-LM is non-polar (there is a symmetry operation that flips it upside down), so they belong to Category I, and the bulk space group is *P*6_3_/*m*2/*m*2/*c* (194), with a single choice of Hall number (488). *n*_c_ = 2 in all these cases (see, *e.g.*, Figure 1a). Table 3 shows that the possible ML-LM point groups are 6̅*m*2 and 3̅m. 6̅*m*2 is for odd *N* (without a center of symmetry) whereas 3̅m occurs for even *N* (with a center of symmetry). We emphasize the assumption *N* ≥ *n*_c_. For graphene (*N* = 1) the point group is 6/*mmm*, meaning that neither of the two *G*_*N*_ occur for *N* < 2 because it has an additional center of symmetry, that disappears in the graphite stacking for any odd *N* > 1.

**Figure 2:**
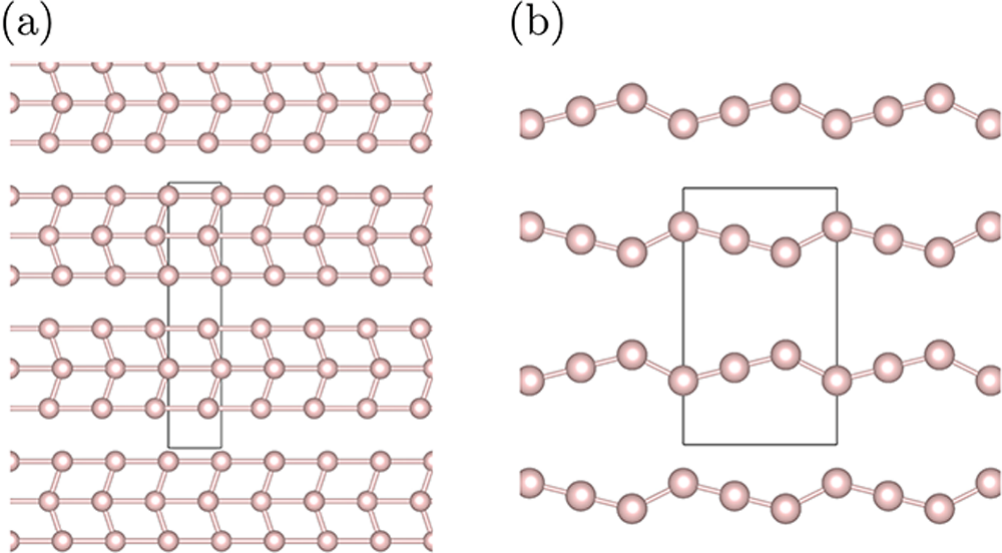
Two fictitious crystals with same B-LM space group (51, Hall number 242, Hall symbol *P*2/*c*2/*m*2_1_/*m*) and *n*_c_ = 2 (with an orthorhombic unit cell and translational invariance in the *y* direction orthogonal to the page). (a) 1L has mirror symmetry, but no inversion. The alternation of point groups for a ML is 2/*m* for even *N*, *mm*2 for odd *N*. (b) 1L has inversion symmetry but no mirror plane. The alternation of point groups for ML is *mm*2 for even *N*, 2/*m* for odd *N*.

From our analysis, it is only possible to identify the set of possible *G*_*N*_ given the Hall number and *n*_c_. To make a specific assignment for odd and even *N*, as in the above example, it is necessary to know the 1L symmetries. To illustrate this, Figure 2a and Figure 2b show two fictitious crystals with the same B-LM space group (51, Hall number 242, Hall symbol *P*2/*c*2/*m*2_1_/*m*) and *n*_c_ = 2. From Table 3, the 2 possibilities for *G*_*N*_ are (i) 2/*m* (having inversion) or (ii) *mm*2 (not having inversion). In both cases, the B-LM has both inversion symmetry and horizontal mirror symmetry, with a corresponding B-LM *G*_b_ = *mmm*. However, 1Ls have either horizontal reflection symmetry (Figure 2a) or inversion symmetry only (Figure 2b). As a result, the inversion and mirror LOC operations have different centers in B-LMs, with the inversion one on middle (layer) planes for Figure 2a (Figure 2b), and horizontal mirror symmetry on layer (middle) planes for Figure 2a (Figure 2b). Because symmetries from middle planes are selected for even *N*, and those from layer planes for odd *N*, for Figure 2a the assignment is 2/*m* for even *N*, and *mm*2 for odd *N*. The opposite holds for Figure 2b.

We now consider some examples from Categories II and III. BiTeCl (Figure 1b) has B-LM space group *P*6_3_*mc* (186, Hall number 480). Because each layer is polar and all have the same polarity (Category II), the point group of any ML-BiTeCl will be *G*_I_ = 3*m* (see Table 3). For Bi_2_TeI (Figure 1c), instead, the B-LM space group is *C*2/*m* (12, Hall number 63). Because it belongs to Category III, the point group of any ML-Bi_2_TeI with odd *N* will be *G*_I_ = *m*. If *N* is even, we then need to check the column for *n*_c_ = 1 in Table 3 (Table 3 can be used by interpreting *n*_c_ as the number of layer pairs, *i.e.*, half of the number of layers in the bulk conventional cell, as discussed in Methods), so that the resulting point group is 2/*m*, independent of the termination of the ML-Bi_2_TeI. Because some entries in Table 3 have two possible values, this implies that, for Category III, some space groups might have an alternation *G*_*N*_ for *N* multiples of 4 or 2, and the specific point group taken will depend on the termination of the finite ML.

### 1.3 Computing Normal Modes

In a fan diagram, we focus only on vibrational modes associated with a rigid relative motion of the layers, typically <100 cm^–1^.

The simplest approximation^26,54,74^ is to model the ML-LM as a finite linear chain of masses with a force constant *K* between them, which might depend on the direction of motion. This is often able to capture the qualitative behavior of the frequency of the modes as a function of *N*, but it might not be able to predict the frequencies or the coupling between C and LB modes accurately in some systems. Extensions of this model have been proposed to include further neighbors^29^ or intralayer coupling,^75^*e.g.*, in the case of MoS_2_, where a diatomic chain model was derived^31^ to take into account the two types of atoms in the system (Mo and S).

Because layers are held together by van der Waals forces (which are typically much weaker than the chemical bonds between atoms in a layer), we derive a more general tensorial model under the following two assumptions: (1) layers move as rigid units, *i.e.*, the atomic displacements **u**(*l*) depend only on the stacking index *l*, and (2) we include only first neighbor interactions between layers. These two assumptions are typically very good in most ML-LMs.^23−25^ In some cases these might break, like at the interface between different or twisted MLs,^29,57^ where further neighbors are needed to fully account for the mode frequencies. Nonetheless, the predictions of our model are still useful to interpret experimental data qualitatively, and could be generalized to include further neighbors, if necessary, within a numerical treatment.

Under these assumptions, the equation of motion can be written as:
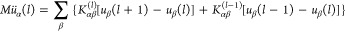
1where *M* is the 1L total mass per unit cell, α and β are Cartesian directions, and *K*_αβ_^(*l*)^ is the (tensorial) force constant between layer *l* and (*l* + 1). Eq 1 is valid for B-LMs when periodic boundary conditions are applied, *u*_β_(*l* = *n*_c_ + 1) = *u*_β_(*l* = 1), and for finite ML-LMs when all *u*_β_(*l*) terms for  < 0 or *l* > *N* are set to zero.

The *K*_αβ_^(*l*)^ tensor, which describes the interaction between two adjacent layers, can be different for each pair of layers. For Category III of Figure 1, there are two types of interfaces that alternate—one set having Te atoms facing each other and the other having Bi atoms—and the corresponding force constants will, thus, be different. Even for Categories I and II, where all layers and interfaces are identical, the matrices for different interfaces between layers *l* and *l* + 1 can differ, *e.g.*, because an interface is obtained from the previous one by a rotation along the vertical axis, or some other symmetry operation (as for MoS_2_ and BiTeCl, see Figure 1a,b). In these cases, the matrices are related by the coincidence operation bringing one layer onto the next one, and we can write  =  = , with *R* the rotational part (proper or improper) of the coincidence operation, and *K* = *K*^(1)^ the interlayer force constant between first and second layer. For Category III, *K*^(*l*)^ can be generated in an analogous way starting from one of the two matrices *K*^(1)^ and *K*^(2)^, depending on the parity of *l*. Thus, in general, we expect not a single *K*_αβ_^(*l*)^, but a set of interlayer force-constant matrices, depending on a few parameters.

In the online tool described in Section 3, we apply and solve numerically eq 1, so we use the appropriately transformed *K*^(*l*)^ for each layer. To get a qualitative understanding of the frequencies, their degeneracies, and their interpretation as C or LB modes, we summarize here the analytical results when there is a single *K*_αβ_ for all layer pairs (*i.e.*, Categories I or II, and the operation *R* commutes with *K*_αβ_^(1)^, so that all *K* matrices are identical). This is the case, *e.g.*, with MoS_2_ or hBN.

Because *K*_αβ_ is symmetrical, it can be diagonalized with eigenvalues *k*_1_, *k*_2_ and *k*_3_. Then, one can solve the equation of motion to get 3*N* solutions (for an ML with *N* layers), obtaining:^26,35^
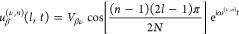
2where *V*_βν_ are the eigenvectors of *K*_αβ_ and ν = 1, 2, 3 denotes three branches (of *N* modes each, indexed by *n* = 1, ..., *N*). The corresponding vibrational frequencies are given by:
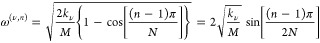
3which can be interpreted as a discretization of the bulk dispersion along the vertical direction at momenta compatible with the finite size of the system.^54,74,76^ The oscillation direction in each branch ν coincides with one of the principal directions of the symmetric tensor, identified by the eigenvector *V*_βν_. In order to define C and LB modes, corresponding, respectively, to oscillations parallel to the layers (in the *xy* plane) and out-of-plane (along *z*), the *K* matrix must be block-diagonal, with a 2×2 block for the C modes and a 1×1 element for the LBM block. In this case, we can then define if ν is a C or LB mode. The frequency of the highest C mode in an ML with *N* layers is usually written as Pos(C)_*N*_ =  = , when expressed in cm^−1^ (with *c* being the speed of light). Similarly Pos(LBM)_*N*_ refers to the highest LBM.

In general, however, the *K* matrix does not have such block form, and the in-plane and out-of-plane vibrations are not decoupled, meaning that a distinction between LB and C modes is not possible, such as in the case of WTe_2_ (see the Methods section for an in-depth discussion on the separation of C and LB modes depending on symmetry).

Because *K* describes the interaction between adjacent layers, its tensorial form (*i.e.*, which elements are zero, which are equal to each other) depends on the crystal system^77^ of the 2L-LM obtained by isolating the two layers, as directly derived from its point group.

The 7 possible cases^77^ (skipping cubic systems, not compatible with a layered structure) are reported in Table 2. For trigonal, hexagonal, and tetragonal 2L-LMs a distinction between LB and C modes is possible and the C branches are degenerate. For orthorhombic systems, LB and C modes can be still defined, but the degeneracy of the two C branches is lifted. For monoclinic systems, we can distinguish two cases: (i) in-plane monoclinic unique axis, for which we can identify one pure C branch, while the other two branches are mixed (no pure LBM can be defined, such as in the case of WTe_2_); (ii) unique axis along the stacking direction, for which we can distinguish LB and C modes, even though the C polarization has no specific orientation with respect to the crystal axes. For triclinic systems, there is no symmetry constraint, therefore a distinction between LB and C modes is not possible (although there might still be a mode mostly polarized orthogonally to the layers, *i.e.*, with a large LB character. This could happen, *e.g.*, in LMHs).

**Table 2:** Components of the *K*_αβ_^(*l*)^ Force-Constants Tensor^a^ According to the Crystal System of the Corresponding 2L-LM Formed by Layers *l* and (*l* + 1)^b^

tetragonal, hexagonal, or trigonal	
orthorhombic	
monoclinic (*y*): in-plane unique axis	
monoclinic (*z*): out-of-plane unique axis	
triclinic	

a From general symmetry considerations, see ref (77).

b The stacking direction is *z*. Non-zero components are indicated. Components that are equal have the same name. For monoclinic systems, we distinguish the case where the unique axis is in-plane (here arbitrarily chosen as *y*), or along *z*.

**Table 3:** Possible ML *G*_*N*_ That Can Be Obtained Knowing the Space Group and Hall Number of the Corresponding B-LM^a^

				ML point group *G*_*N*_					
Hall number	bulk space group	*G*_b_	*G*_I_	*n*_c_ = 1	*n*_c_ = 2	*n*_c_ = 3	*n*_c_ = 4	*n*_c_ = 5	*n*_c_ = 6
	**Triclinic**								
1	1 (*P*1)	1	1	1	1	1	1	1	1
2	2 (*P*1̅)	1̅	1	1̅	1 or 1̅	×	×	×	×

	**Monoclinic**								
3	3 (*P*121)	2	1	2	1 or 2	×	×	×	×
4	3 (*P*112)	2	2	2	2	2	2	2	2
*Equivalent Hall numbers:*		5 (*P*211) → 3							
6	4 (*P*12_1_1)	2	1	2	1 or 2	×	×	×	×
7	4 (*P*112_1_)	2	1	/	1	/	1	/	1
*Equivalent Hall numbers:*		8 (*P*2_1_11) → 6							
9	5 (*C*121)	2	1	2	1 or 2	×	×	×	×
11	5 (*I*121)	2	1	/	2	/	1 or 2	/	×
12	5 (*A*112)	2	2	/	2	/	2	/	2
*Equivalent Hall numbers:*		10 (*A*121) → 11, 13 (*B*112) → 12, 14 (*I*112) → 12,15 (*B*211) → 11, 16 (*C*211) → 9, 17 (*I*211) → 11							
18	6 (*P*1*m*1)	*m*	*m*	*m*	*m*	*m*	*m*	*m*	*m*
19	6 (*P*11*m*)	*m*	1	*m*	1 or *m*	×	×	×	×
*Equivalent Hall numbers:*		20 (*Pm*11) → 18							
21	7 (*P*1*c*1)	*m*	1	/	1	/	1	/	1
23	7 (*P*1*a*1)	*m*	*m*	*m*	*m*	*m*	*m*	*m*	*m*
24	7 (*P*11*a*)	*m*	1	*m*	1 or *m*	×	×	×	×
*Equivalent Hall numbers:*		22 (*P*1*n*1) → 21, 25 (*P*11*n*) → 24, 26 (*P*11*b*) → 24, 27 (*Pb*11) → 23, 28 (*Pn*11) → 21, 29 (*Pc*11) → 21							
30	8 (*C*1*m*1)	*m*	*m*	*m*	*m*	*m*	*m*	*m*	*m*
32	8 (*I*1*m*1)	*m*	*m*	/	*m*	/	*m*	/	*m*
33	8 (*A*11*m*)	*m*	1	/	*m*	/	1 or *m*	/	×
*Equivalent Hall numbers:*		31 (*A*1*m*1) → 32, 34 (*B*11*m*) → 33, 35 (*I*11*m*) → 33, 36 (*Bm*11) → 32, 37 (*Cm*11) → 30, 38 (*Im*11) → 32							
39	9 (*C*1*c*1)	*m*	1	/	1	/	1	/	1
41	9 (*I*1*a*1)	*m*	*m*	/	*m*	/	*m*	/	*m*
45	9 (*A*11*a*)	*m*	1	/	*m*	/	1 or *m*	/	×
*Equivalent Hall numbers:*		40 (*A*1*n*1) → 41, 42 (*A*1*a*1) → 41, 43 (*C*1*n*1) → 39, 44 (*I*1*c*1) → 41, 46 (*B*11*n*) → 45, 47 (*I*11*b*) → 45, 48 (*B*11*b*) → 45, 49 (*A*11*n*) → 45, 50 (*I*11*a*) → 45, 51 (*Bb*11) → 41, 52 (*Cn*11) → 39, 53 (*Ic*11) → 41, 54 (*Cc*11) → 39, 55 (*Bn*11) → 41, 56 (*Ib*11) → 41							
57	10 (*P*12/*m*1)	2/*m*	*m*	2/*m*	2/*m* or *m*	×	×	×	×
58	10 (*P*112/*m*)	2/*m*	2	2/*m*	2 or 2/*m*	×	×	×	×
*Equivalent Hall numbers:*		59 (*P*2/*m*11) → 57							
60	11 (*P*12_1_/*m*1)	2/*m*	*m*	2/*m*	2/*m* or *m*	×	×	×	×
61	11 (*P*112_1_/*m*)	2/*m*	1	/	1̅ or *m*	/	×	/	×
*Equivalent Hall numbers:*		62 (*P*2_1_/*m*11) → 60							
63	12 (*C*12/*m*1)	2/*m*	*m*	2/*m*	2/*m* or *m*	×	×	×	×
65	12 (*I*12/*m*1)	2/*m*	*m*	/	2/*m*	/	2/*m* or *m*	/	×
66	12 (*A*112/*m*)	2/*m*	2	/	2/*m*	/	2 or 2/*m*	/	×
*Equivalent Hall numbers:*		64 (*A*12/*m*1) → 65, 67 (*B*112/*m*) → 66, 68 (*I*112/*m*) → 66, 69 (*B*2/*m*11) → 65, 70 (*C*2/*m*11) → 63, 71 (*I*2/*m*11) → 65							
72	13 (*P*12/*c*1)	2/*m*	1	/	2 or 1̅	/	×	/	×
74	13 (*P*12/*a*1)	2/*m*	*m*	2/*m*	2/*m* or *m*	×	×	×	×
75	13 (*P*112/*a*)	2/*m*	2	2/*m*	2 or 2/*m*	×	×	×	×
*Equivalent Hall numbers:*		73 (*P*12/*n*1) → 72, 76 (*P*112/*n*) → 75, 77 (*P*112/*b*) → 75, 78 (*P*2/*b*11) → 74, 79 (*P*2/*n*11) → 72, 80 (*P*2/*c*11) → 72							
81	14 (*P*12_1_/*c*1)	2/*m*	1	/	2 or 1̅	/	×	/	×
83	14 (*P*12_1_/*a*1)	2/*m*	*m*	2/*m*	2/*m* or *m*	×	×	×	×
84	14 (*P*112_1_/*a*)	2/*m*	1	/	1̅ or *m*	/	×	/	×
*Equivalent Hall numbers:*		82 (*P*12_1_/*n*1) → 81, 85 (*P*112_1_/n) → 84, 86 (*P*112_1_/*b*) → 84, 87 (*P*2_1_/*b*11) → 83, 88 (*P*2_1_/*n*11) → 81, 89 (*P*2_1_/*c*11) → 81							
90	15 (*C*12/*c*1)	2/*m*	1	/	2 or 1̅	/	×	/	×
92	15 (*I*12/*a*1)	2/*m*	*m*	/	2/*m*	/	2/*m* or *m*	/	×
96	15 (*A*112/*a*)	2/*m*	2	/	2/*m*	/	2 or 2/*m*	/	×
*Equivalent Hall numbers:*		91 (*A*12/*n*1) → 92, 93 (*A*12/*a*1) → 92, 94 (*C*12/*n*1) → 90, 95 (*I*12/*c*1) → 92, 97 (*B*112/*n*) → 96, 98 (*I*112/*b*) → 96, 99 (*B*112/*b*) → 96, 100 (*A*112/*n*) → 96, 101 (*I*112/*a*) → 96, 102 (*B*2/*b*11) → 92, 103 (*C*2/*n*11) → 90, 104 (*I*2/*c*11) → 92, 105 (*C*2/*c*11) → 90, 106 (*B*2/*n*11) → 92, 107 (*I*2/*b*11) → 92							

	**Orthorhombic**								
108	16 (*P*222)	222	2	222	2 or 222	×	×	×	×
109	17 (*P*222_1_)	222	1	/	2	/	1 or 2	/	×
110	17 (*P*2_1_22)	222	2	222	2 or 222	×	×	×	×
*Equivalent Hall numbers:*		111 (*P*22_1_2) → 110							
112	18 (*P*2_1_2_1_2)	222	2	222	2 or 222	×	×	×	×
113	18 (*P*22_1_2_1_)	222	1	/	2	/	1 or 2	/	×
*Equivalent Hall numbers:*		114 (*P*2_1_22_1_) → 113							
115	19 (*P*2_1_2_1_2_1_)	222	1	/	2	/	1 or 2	/	×
116	20 (*C*222_1_)	222	1	/	2	/	1 or 2	/	×
117	20 (*A*2_1_22)	222	2	/	222	/	2 or 222	/	×
*Equivalent Hall numbers:*		118 (*B*22_1_2) → 117							
119	21 (*C*222)	222	2	222	2 or 222	×	×	×	×
120	21 (*A*222)	222	2	/	222	/	2 or 222	/	×
*Equivalent Hall numbers:*		121 (*B*222) → 120							
122	22 (*F*222)	222	2	/	222	/	2 or 222	/	×
123	23 (*I*222)	222	2	/	222	/	2 or 222	/	×
124	24 (*I*2_1_2_1_2_1_)	222	2	/	222	/	2 or 222	/	×
125	25 (*Pmm*2)	*mm*2	*mm*2	*mm*2	*mm*2	*mm*2	*mm*2	*mm*2	*mm*2
126	25 (*P*2*mm*)	*mm*2	*m*	*mm*2	*m* or *mm*2	×	×	×	×
*Equivalent Hall numbers:*		127 (*Pm*2*m*) → 126							
128	26 (*Pmc*2_1_)	*mm*2	*m*	/	*m*	/	*m*	/	*m*
130	26 (*P*2_1_*ma*)	*mm*2	*m*	*mm*2	*m* or *mm*2	×	×	×	×
*Equivalent Hall numbers:*		129 (*Pcm*2_1_) → 128, 131 (*P*2_1_*am*) → 130, 132 (*Pb*2_1_*m*) → 130, 133 (*Pm*2_1_*b*) → 130							
134	27 (*Pcc*2)	*mm*2	2	/	2	/	2	/	2
135	27 (*P*2*aa*)	*mm*2	*m*	*mm*2	*m* or *mm*2	×	×	×	×
*Equivalent Hall numbers:*		136 (*Pb*2*b*) → 135							
137	28 (*Pma*2)	*mm*2	*mm*2	*mm*2	*mm*2	*mm*2	*mm*2	*mm*2	*mm*2
139	28 (*P*2*mb*)	*mm*2	*m*	*mm*2	*m* or *mm*2	×	×	×	×
140	28 (*P*2*cm*)	*mm*2	1	/	2 or *m*	/	×	/	×
*Equivalent Hall numbers:*		138 (*Pbm*2) → 137, 141 (*Pc*2*m*) → 140, 142 (*Pm*2*a*) → 139							
143	29 (*Pca*2_1_)	*mm*2	*m*	/	*m*	/	*m*	/	*m*
145	29 (*P*2_1_*ab*)	*mm*2	*m*	*mm*2	*m* or *mm*2	×	×	×	×
146	29 (*P*2_1_*ca*)	*mm*2	1	/	2 or *m*	/	×	/	×
*Equivalent Hall numbers:*		144 (*Pbc*2_1_) → 143, 147 (*Pc*2_1_*b*) → 146, 148 (*Pb*2_1_*a*) → 145							
149	30 (*Pnc*2)	*mm*2	2	/	2	/	2	/	2
151	30 (*P*2*na*)	*mm*2	1	/	2 or *m*	/	×	/	×
152	30 (*P*2*an*)	*mm*2	*m*	*mm*2	*m* or *mm*2	×	×	×	×
*Equivalent Hall numbers:*		150 (*Pcn*2) → 149, 153 (*Pb*2*n*) → 152, 154 (*Pn*2*b*) → 151							
155	31 (*Pmn*2_1_)	*mm*2	*m*	/	*m*	/	*m*	/	*m*
157	31 (*P*2_1_*mn*)	*mm*2	*m*	*mm*2	*m* or *mm*2	×	×	×	×
158	31 (*P*2_1_*nm*)	*mm*2	1	/	2 or *m*	/	×	/	×
*Equivalent Hall numbers:*		156 (*Pnm*2_1_) → 155, 159 (*Pn*2_1_*m*) → 158, 160 (*Pm*2_1_*n*) → 157							
161	32 (*Pba*2)	*mm*2	*mm*2	*mm*2	*mm*2	*mm*2	*mm*2	*mm*2	*mm*2
162	32 (*P*2*cb*)	*mm*2	1	/	2 or *m*	/	×	/	×
*Equivalent Hall numbers:*		163 (*Pc*2*a*) → 162							
164	33 (*Pna*2_1_)	*mm*2	*m*	/	*m*	/	*m*	/	*m*
166	33 (*P*2_1_*nb*)	*mm*2	1	/	2 or *m*	/	×	/	×
*Equivalent Hall numbers:*		165 (*Pbn*2_1_) → 164, 167 (*P*2_1_cn) → 166, 168 (*Pc*2_1_*n*) → 166, 169 (*Pn*2_1_*a*) → 166							
170	34 (*Pnn*2)	*mm*2	2	/	2	/	2	/	2
171	34 (*P*2*nn*)	*mm*2	1	/	2 or *m*	/	×	/	×
*Equivalent Hall numbers:*		172 (*Pn*2*n*) → 171							
173	35 (*Cmm*2)	*mm*2	*mm*2	*mm*2	*mm*2	*mm*2	*mm*2	*mm*2	*mm*2
174	35 (*A*2*mm*)	*mm*2	*m*	/	*mm*2	/	*m* or *mm*2	/	×
*Equivalent Hall numbers:*		175 (*Bm*2*m*) → 174							
176	36 (*Cmc*2_1_)	*mm*2	*m*	/	*m*	/	*m*	/	*m*
178	36 (*A*2_1_*ma*)	*mm*2	*m*	/	*mm*2	/	*m* or *mm*2	/	×
*Equivalent Hall numbers:*		177 (*Ccm*2_1_) → 176, 179 (*A*2_1_*am*) → 178, 180 (*Bb*2_1_*m*) → 178, 181 (*Bm*2_1_*b*) → 178							
182	37 (*Ccc*2)	*mm*2	2	/	2	/	2	/	2
183	37 (*A*2*aa*)	*mm*2	*m*	/	*mm*2	/	*m* or *mm*2	/	×
*Equivalent Hall numbers:*		184 (*Bb*2*b*) → 183							
185	38 (*Amm*2)	*mm*2	*mm*2	/	*mm*2	/	*mm*2	/	*mm*2
187	38 (*B*2*mm*)	*mm*2	*m*	/	*mm*2	/	*m* or *mm*2	/	×
188	38 (*C*2*mm*)	*mm*2	*m*	*mm*2	*m* or *mm*2	×	×	×	×
*Equivalent Hall numbers:*		186 (*Bmm*2) → 185, 189 (*Cm*2*m*) → 188, 190 (*Am*2*m*) → 187							
191	39 (*Abm*2)	*mm*2	*mm*2	/	*mm*2	/	*mm*2	/	*mm*2
193	39 (*B*2*cm*)	*mm*2	*m*	/	*mm*2	/	*m* or *mm*2	/	×
194	39 (*C*2*mb*)	*mm*2	*m*	*mm*2	*m* or *mm*2	×	×	×	×
*Equivalent Hall numbers:*		192 (*Bma*2) → 191, 195 (*Cm*2*a*) → 194, 196 (A*c*2*m*) → 193							
197	40 (*Ama*2)	*mm*2	*mm*2	/	*mm*2	/	*mm*2	/	*mm*2
199	40 (*B*2*mb*)	*mm*2	*m*	/	*mm*2	/	*m* or *mm*2	/	×
200	40 (*C*2*cm*)	*mm*2	1	/	2 or *m*	/	×	/	×
*Equivalent Hall numbers:*		198 (*Bbm*2) → 197, 201 (*Cc*2*m*) → 200, 202 (*Am*2*a*) → 199							
203	41 (*Aba*2)	*mm*2	*mm*2	/	*mm*2	/	*mm*2	/	*mm*2
205	41 (*B*2*cb*)	*mm*2	*m*	/	*mm*2	/	*m* or *mm*2	/	×
206	41 (*C*2*cb*)	*mm*2	1	/	2 or *m*	/	×	/	×
*Equivalent Hall numbers:*		204 (*Bba*2) → 203, 207 (*Cc*2*a*) → 206, 208 (*Ac*2*a*) → 205							
209	42 (*Fmm*2)	*mm*2	*mm*2	/	*mm*2	/	*mm*2	/	*mm*2
210	42 (*F*2*mm*)	*mm*2	*m*	/	*mm*2	/	*m* or *mm*2	/	×
*Equivalent Hall numbers:*		211 (*Fm*2*m*) → 210							
212	43 (*Fdd*2)	*mm*2	2	/	/	/	2	/	/
213	43 (*F*2*dd*)	*mm*2	1	/	/	/	2 or *m*	/	/
*Equivalent Hall numbers:*		214 (*Fd*2*d*) → 213							
215	44 (*Imm*2)	*mm*2	*mm*2	/	*mm*2	/	*mm*2	/	*mm*2
216	44 (*I*2*mm*)	*mm*2	*m*	/	*mm*2	/	*m* or *mm*2	/	×
*Equivalent Hall numbers:*		217 (*Im*2*m*) → 216							
218	45 (*Iba*2)	*mm*2	*mm*2	/	*mm*2	/	*mm*2	/	*mm*2
219	45 (*I*2cb)	*mm*2	*m*	/	*mm*2	/	*m* or *mm*2	/	×
*Equivalent Hall numbers:*		220 (*Ic*2*a*) → 219							
221	46 (*Ima*2)	*mm*2	*mm*2	/	*mm*2	/	*mm*2	/	*mm*2
223	46 (*I*2*mb*)	*mm*2	*m*	/	*mm*2	/	*m* or *mm*2	/	×
*Equivalent Hall numbers:*		222 (*Ibm*2) → 221, 224 (*I*2*cm*) → 223, 225 (*Ic*2*m*) → 223, 226 (*Im*2*a*) → 223							
227	47 (*P*2/*m*2/*m*2/*m*)	*mmm*	*mm*2	*mmm*	*mm*2 or *mmm*	×	×	×	×
228	48 (*P*2/*n*2/*n*2/*n*)	*mmm*	2	/	2/*m* or 222	/	×	/	×
*Equivalent Hall numbers:*		229 (*P*2/*n*2/*n*2/*n*) → 228							
230	49 (*P*2/*c*2/*c*2/*m*)	*mmm*	2	/	2/*m* or 222	/	×	/	×
231	49 (*P*2/*m*2/*a*2/*a*)	*mmm*	*mm*2	*mmm*	*mm*2 or *mmm*	×	×	×	×
*Equivalent Hall numbers:*		232 (*P*2/*b*2/*m*2/*b*) → 231							
233	50 (*P*2/*b*2/*a*2/*n*)	*mmm*	*mm*2	*mmm*	*mm*2 or *mmm*	×	×	×	×
235	50 (*P*2/*n*2/*c*2/*b*)	*mmm*	2	/	2/*m* or 222	/	×	/	×
*Equivalent Hall numbers:*		234 (*P*2/*b*2/*a*2/*n*) → 233, 236 (*P*2/*n*2/*c*2/*b*) → 235, 237 (*P*2/*c*2/*n*2/*a*) → 235, 238 (*P*2/*c*2/*n*2/*a*) → 235							
239	51 (*P*2_1_/*m*2/*m*2/*a*)	*mmm*	*mm*2	*mmm*	*mm*2 or *mmm*	×	×	×	×
242	51 (*P*2/*c*2/*m*2_1_/*m*)	*mmm*	*m*	/	2/*m* or *mm*2	/	×	/	×
*Equivalent Hall numbers:*		240 (*P*2/*m*2_1_/*m*2/*b*) → 239, 241 (*P*2/*b*2_1_/*m*2/*m*) → 239, 243 (*P*2/*m*2/*c*2_1_/*m*) → 242, 244 (*P*2_1_/*m*2/*a*2/*m*) → 239							
245	52 (*P*2/*n*2_1_/*n*2/*a*)	*mmm*	2	/	2/*m* or 222	/	×	/	×
247	52 (*P*2/*b*2/*n*2_1_/*n*)	*mmm*	*m*	/	2/*m* or *mm*2	/	×	/	×
*Equivalent Hall numbers:*		246 (*P*2_1_/*n*2/*n*2/*b*) → 245, 248 (*P*2/*c*2_1_/*n*2/*n*) → 245, 249 (*P*2_1_/*n*2/*c*2/*n*) → 245, 250 (*P*2/*n*2/*a*2_1_/*n*) → 247							
251	53 (*P*2/*m*2/*n*2_1_/*a*)	*mmm*	*m*	/	2/*m* or *mm*2	/	×	/	×
253	53 (*P*2_1_/*b*2/*m*2/*n*)	*mmm*	*mm*2	*mmm*	*mm*2 or *mmm*	×	×	×	×
254	53 (*P*2_1_/*c*2/*n*2/*m*)	*mmm*	2	/	2/*m* or 222	/	×	/	×
*Equivalent Hall numbers:*		252 (*P*2/*n*2/*m*2_1_/*b*) → 251, 255 (*P*2/*n*2_1_/*c*2/*m*) → 254, 256 (*P*2/*m*2_1_/*a*2/*n*) → 253							
257	54 (*P*2_1_/*c*2/*c*2/*a*)	*mmm*	2	/	2/*m* or 222	/	×	/	×
259	54 (*P*2/*b*2_1_/*a*2/*a*)	*mmm*	*mm*2	*mmm*	*mm*2 or *mmm*	×	×	×	×
260	54 (*P*2/*c*2/*a*2_1_/*a*)	*mmm*	*m*	/	2/*m* or *mm*2	/	×	/	×
*Equivalent Hall numbers:*		258 (*P*2/*c*2_1_/*c*2/*b*) → 257, 261 (*P*2/*b*2/*c*2_1_/*b*) → 260, 262 (*P*2_1_/*b*2/*a*2/*b*) → 259							
263	55 (*P*2_1_/*b*2_1_/*a*2/*m*)	*mmm*	*mm*2	*mmm*	*mm*2 or *mmm*	×	×	×	×
264	55 (*P*2/*m*2_1_/*c*2_1_/*b*)	*mmm*	*m*	/	2/*m* or *mm*2	/	×	/	×
*Equivalent Hall numbers:*		265 (*P*2_1_/*c*2/*m*2_1_/*a*) → 264							
266	56 (*P*2_1_/*c*2_1_/*c*2/*n*)	*mmm*	2	/	2/*m* or 222	/	×	/	×
267	56 (*P*2/*n*2_1_/*a*2_1_/*a*)	*mmm*	*m*	/	2/*m* or *mm*2	/	×	/	×
*Equivalent Hall numbers:*		268 (*P*2_1_/*b*2/*n*2_1_/*b*) → 267							
269	57 (*P*2/*b*2_1_/*c*2_1_/*m*)	*mmm*	*m*	/	2/*m* or *mm*2	/	×	/	×
272	57 (*P*2_1_/*m*2_1_/*a*2/*b*)	*mmm*	*mm*2	*mmm*	*mm*2 or *mmm*	×	×	×	×
*Equivalent Hall numbers:*		270 (*P*2_1_/*c*2/*a*2_1_/*m*) → 269, 271 (*P*2_1_/*m*2/*c*2_1_/*a*) → 269, 273 (*P*2_1_/*b*2_1_/*m*2/*a*) → 272, 274 (*P*2/*c*2_1_/*m*2_1_/*b*) → 269							
275	58 (*P*2_1_/*n*2_1_/*n*2/*m*)	*mmm*	2	/	2/*m* or 222	/	×	/	×
276	58 (*P*2/*m*2_1_/*n*2_1_/*n*)	*mmm*	*m*	/	2/*m* or *mm*2	/	×	/	×
*Equivalent Hall numbers:*		277 (*P*2_1_/*n*2/*m*2_1_/*n*) → 276							
278	59 (*P*2_1_/*m*2_1_/*m*2/*n*)	*mmm*	*mm*2	*mmm*	*mm*2 or *mmm*	×	×	×	×
280	59 (*P*2/*n*2_1_/*m*2_1_/*m*)	*mmm*	*m*	/	2/*m* or *mm*2	/	×	/	×
*Equivalent Hall numbers:*		279 (*P*2_1_/*m*2_1_/*m*2/*n*) → 278, 281 (*P*2/*n*2_1_/*m*2_1_/*m*) → 280, 282 (*P*2_1_/*m*2/*n*2_1_/*m*) → 280, 283 (*P*2_1_/*m*2/*n*2_1_/*m*) → 280							
284	60 (*P*2_1_/*b*2/*c*2_1_/*n*)	*mmm*	*m*	/	2/*m* or *mm*2	/	×	/	×
286	60 (*P*2_1_/*n*2_1_/*c*2/*a*)	*mmm*	2	/	2/*m* or 222	/	×	/	×
*Equivalent Hall numbers:*		285 (*P*2/*c*2_1_/*a*2_1_/*n*) → 284, 287 (*P*2_1_/*n*2/*a*2_1_/*b*) → 284, 288 (*P*2/*b*2_1_/*n*2_1_/*a*) → 284, 289 (*P*2_1_/*c*2_1_/*n*2/*b*) → 286							
290	61 (*P*2_1_/*b*2_1_/*c*2_1_/*a*)	*mmm*	*m*	/	2/*m* or *mm*2	/	×	/	×
*Equivalent Hall numbers:*		291 (*P*2_1_/*c*2_1_/*a*2_1_/*b*) → 290							
292	62 (*P*2_1_/*n*2_1_/*m*2_1_/*a*)	*mmm*	*m*	/	2/*m* or *mm*2	/	×	/	×
*Equivalent Hall numbers:*		293 (*P*2_1_/*m*2_1_/*n*2_1_/*b*) → 292, 294 (*P*2_1_/*b*2_1_/*n*2_1_/*m*) → 292, 295 (*P*2_1_/*c*2_1_/*m*2_1_/*n*) → 292, 296 (*P*2_1_/*m*2_1_/*c*2_1_/*n*) → 292, 297 (*P*2_1_/*n*2_1_/*a*2_1_/*m*) → 292							
298	63 (*C*2/*m*2/*c*2_1_/*m*)	*mmm*	*m*	/	2/*m* or *mm*2	/	×	/	×
300	63 (*A*2_1_/*m*2/*m*2/*a*)	*mmm*	*mm*2	/	*mmm*	/	*mm*2 or *mmm*	/	×
*Equivalent Hall numbers:*		299 (*C*2/*c*2/*m*2_1_/*m*) → 298, 301 (*A*2_1_/*m*2/*a*2/*m*) → 300, 302 (*B*2/*b*2_1_/*m*2/*m*) → 300, 303 (*B*2/*m*2_1_/*m*2/*b*) → 300							
304	64 (*C*2/*m*2/*c*2_1_/*a*)	*mmm*	*m*	/	2/*m* or *mm*2	/	×	/	×
306	64 (*A*2_1_/*b*2/*m*2/*a*)	*mmm*	*mm*2	/	*mmm*	/	*mm*2 or *mmm*	/	×
*Equivalent Hall numbers:*		305 (*C*2/*c*2/*m*2_1_/*b*) → 304, 307 (*A*2_1_/*c*2/*a*2/*m*) → 306, 308 (*B*2/*b*2_1_/*c*2/*m*) → 306, 309 (*B*2/*m*2_1_/*a*2/*b*) → 306							
310	65 (*C*2/*m*2/*m*2/*m*)	*mmm*	*mm*2	*mmm*	*mm*2 or *mmm*	×	×	×	×
311	65 (*A*2/*m*2/*m*2/*m*)	*mmm*	*mm*2	/	*mmm*	/	*mm*2 or *mmm*	/	×
*Equivalent Hall numbers:*		312 (*B*2/*m*2/*m*2/*m*) → 311							
313	66 (*C*2/*c*2/*c*2/*m*)	*mmm*	2	/	2/*m* or 222	/	×	/	×
314	66 (*A*2/*m*2/*a*2/*a*)	*mmm*	*mm*2	/	*mmm*	/	*mm*2 or *mmm*	/	×
*Equivalent Hall numbers:*		315 (*B*2/*b*2/*m*2/*b*) → 314							
316	67 (*C*2/*m*2/*m*2/*a*)	*mmm*	*mm*2	*mmm*	*mm*2 or *mmm*	×	×	×	×
318	67 (*A*2/*b*2/*m*2/*m*)	*mmm*	*mm*2	/	*mmm*	/	*mm*2 or *mmm*	/	×
*Equivalent Hall numbers:*		317 (*C*2/*m*2/*m*2/*b*) → 316, 319 (*A*2/*c*2/*m*2/*m*) → 318, 320 (*B*2/*m*2/*c*2/*m*) → 318, 321 (*B*2/*m*2/*a*2/*m*) → 318							
322	68 (*C*2/*c*2/*c*2/*a*)	*mmm*	2	/	2/*m* or 222	/	×	/	×
326	68 (*A*2/*b*2/*a*2/*a*)	*mmm*	*mm*2	/	*mmm*	/	*mm*2 or *mmm*	/	×
*Equivalent Hall numbers:*		323 (*C*2/*c*2/*c*2/*a*) → 322, 324 (*C*2/*c*2/*c*2/*b*) → 322, 325 (*C*2/*c*2/*c*2/*b*) → 322, 327 (*A*2/*b*2/*a*2/*a*) → 326, 328 (*A*2/*c*2/*a*2/*a*) → 326, 329 (*A*2/*c*2/*a*2/*a*) → 326, 330 (*B*2/*b*2/*c*2/*b*) → 326, 331 (*B*2/*b*2/*c*2/*b*) → 326, 332 (*B*2/*b*2/*a*2/*b*) → 326, 333 (*B*2/*b*2/*a*2/*b*) → 326							
334	69 (*F*2/*m*2/*m*2/*m*)	*mmm*	*mm*2	/	*mmm*	/	*mm*2 or *mmm*	/	×
335	70 (*F*2/*d*2/*d*2/*d*)	*mmm*	2	/	/	/	2/*m* or 222	/	/
*Equivalent Hall numbers:*		336 (*F*2/*d*2/*d*2/*d*) → 335							
337	71 (*I*2/*m*2/*m*2/*m*)	*mmm*	*mm*2	/	*mmm*	/	*mm*2 or *mmm*	/	×
338	72 (*I*2/*b*2/*a*2/*m*)	*mmm*	*mm*2	/	*mmm*	/	*mm*2 or *mmm*	/	×
*Equivalent Hall numbers:*		339 (*I*2/*m*2/*c*2/*b*) → 338, 340 (*I*2/*c*2/*m*2/*a*) → 338							
341	73 (*I*2/*b*2/*c*2/*a*)	*mmm*	*mm*2	/	*mmm*	/	*mm*2 or *mmm*	/	×
*Equivalent Hall numbers:*		342 (*I*2/*c*2/*a*2/*b*) → 341							
343	74 (*I*2/*m*2/*m*2/*a*)	*mmm*	*mm*2	/	*mmm*	/	*mm*2 or *mmm*	/	×
*Equivalent Hall numbers:*		344 (*I*2/*m*2/*m*2/*b*) → 343, 345 (*I*2/*b*2/*m*2/*m*) → 343, 346 (*I*2/*c*2/*m*2/*m*) → 343, 347 (*I*2/*m*2/*c*2/*m*) → 343, 348 (*I*2/*m*2/*a*2/*m*) → 343							

	**Tetragonal**								
349	75 (*P*4)	4	4	4	4	4	4	4	4
350	76 (*P*4_1_)	4	1	/	/	/	1	/	/
351	77 (*P*4_2_)	4	2	/	2	/	2	/	2
352	78 (*P*4_3_)	4	1	/	/	/	1	/	/
353	79 (*I*4)	4	4	/	4	/	4	/	4
354	80 (*I*4_1_)	4	2	/	/	/	2	/	/
355	81 (*P*4̅)	4̅	2	4̅	2 or 4̅	×	×	×	×
356	82 (*I*4̅)	4̅	2	/	4̅	/	2 or 4̅	/	×
357	83 (*P*4/*m*)	4/*m*	4	4/*m*	4 or 4/*m*	×	×	×	×
358	84 (*P*4_2_/*m*)	4/*m*	2	/	2/*m* or 4̅	/	×	/	×
359	85 (*P*4/*n*)	4/*m*	4	4/*m*	4 or 4/*m*	×	×	×	×
*Equivalent Hall numbers:*		360 (*P*4/*n*) → 359							
361	86 (*P*4_2_/*n*)	4/*m*	2	/	2/*m* or 4̅	/	×	/	×
*Equivalent Hall numbers:*		362 (*P*4_2_/*n*) → 361							
363	87 (*I*4/*m*)	4/*m*	4	/	4/*m*	/	4 or 4/*m*	/	×
364	88 (*I*4_1_/*a*)	4/*m*	2	/	/	/	2/*m* or 4̅	/	/
*Equivalent Hall numbers:*		365 (*I*4_1_/*a*) → 364							
366	89 (*P*422)	422	4	422	4 or 422	×	×	×	×
367	90 (*P*42_1_2)	422	4	422	4 or 422	×	×	×	×
368	91 (*P*4_1_22)	422	1	/	/	/	2	/	/
369	92 (*P*4_1_2_1_2)	422	1	/	/	/	2	/	/
370	93 (*P*4_2_22)	422	2	/	222	/	2 or 222	/	×
371	94 (*P*4_2_2_1_2)	422	2	/	222	/	2 or 222	/	×
372	95 (*P*4_3_22)	422	1	/	/	/	2	/	/
373	96 (*P*4_3_2_1_2)	422	1	/	/	/	2	/	/
374	97 (*I*422)	422	4	/	422	/	4 or 422	/	×
375	98 (*I*4_1_22)	422	2	/	/	/	222	/	/
376	99 (*P*4*mm*)	4*mm*	4*mm*	4*mm*	4*mm*	4*mm*	4*mm*	4*mm*	4*mm*
377	100 (*P*4*bm*)	4*mm*	4*mm*	4*mm*	4*mm*	4*mm*	4*mm*	4*mm*	4*mm*
378	101 (*P*4_2_*cm*)	4*mm*	*mm*2	/	*mm*2	/	*mm*2	/	*mm*2
379	102 (*P*4_2_*nm*)	4*mm*	*mm*2	/	*mm*2	/	*mm*2	/	*mm*2
380	103 (*P*4*cc*)	4*mm*	4	/	4	/	4	/	4
381	104 (*P*4*nc*)	4*mm*	4	/	4	/	4	/	4
382	105 (*P*4_2_*mc*)	4*mm*	*mm*2	/	*mm*2	/	*mm*2	/	*mm*2
383	106 (*P*4_2_*bc*)	4*mm*	*mm*2	/	*mm*2	/	*mm*2	/	*mm*2
384	107 (*I*4*mm*)	4*mm*	4*mm*	/	4*mm*	/	4*mm*	/	4*mm*
385	108 (*I*4*cm*)	4*mm*	4*mm*	/	4*mm*	/	4*mm*	/	4*mm*
386	109 (*I*4_1_*md*)	4*mm*	*mm*2	/	/	/	*mm*2	/	/
387	110 (*I*4_1_*cd*)	4*mm*	*mm*2	/	/	/	*mm*2	/	/
388	111 (*P*4̅2*m*)	4̅2*m*	*mm*2	4̅2*m*	4̅2*m* or *mm*2	×	×	×	×
389	112 (*P*4̅2c)	4̅2*m*	2	/	222 or 4̅	/	×	/	×
390	113 (*P*4̅2_1_*m*)	4̅2*m*	*mm*2	4̅2*m*	4̅2*m* or *mm*2	×	×	×	×
391	114 (*P*4̅2_1_c)	4̅2*m*	2	/	222 or 4̅	/	×	/	×
392	115 (*P*4̅*m*2)	4̅2*m*	*mm*2	4̅2*m*	4̅2*m* or *mm*2	×	×	×	×
393	116 (*P*4̅*c*2)	4̅2*m*	2	/	222 or 4̅	/	×	/	×
394	117 (*P*4̅*b*2)	4̅2*m*	*mm*2	4̅2*m*	4̅2*m* or *mm*2	×	×	×	×
395	118 (*P*4̅*n*2)	42̅*m*	2	/	222 or 4̅	/	×	/	×
396	119 (*I*4̅*m*2)	4̅2*m*	*mm*2	/	4̅2*m*	/	4̅2*m* or *mm*2	/	×
397	120 (*I*4̅*c*2)	4̅2*m*	*mm*2	/	4̅2*m*	/	4̅2*m* or *mm*2	/	×
398	121 (*I*4̅2*m*)	4̅2*m*	*mm*2	/	4̅2*m*	/	4̅2*m* or *mm*2	/	×
399	122 (*I*4̅2d)	4̅2*m*	2	/	/	/	222 or 4̅	/	/
400	123 (*P*4/*m*2/*m*2/*m*)	4/*mmm*	4*mm*	4/*mmm*	4*mm* or 4/*mmm*	×	×	×	×
401	124 (*P*4/*m*2/*c*2/*c*)	4/*mmm*	4	/	4/*m* or 422	/	×	/	×
402	125 (*P*4/*n*2/*b*2/*m*)	4/*mmm*	4*mm*	4/*mmm*	4*mm* or 4/*mmm*	×	×	×	×
*Equivalent Hall numbers:*		403 (*P*4/*n*2/*b*2/*m*) → 402							
404	126 (*P*4/*n*2/*n*2/*c*)	4/*mmm*	4	/	4/*m* or 422	/	×	/	×
*Equivalent Hall numbers:*		405 (*P*4/*n*2/*n*2/*c*) → 404							
406	127 (*P*4/*m*2_1_/*bm*)	4/*mmm*	4*mm*	4/*mmm*	4*mm* or 4/*mmm*	×	×	×	×
407	128 (*P*4/*m*2_1_/*nc*)	4/*mmm*	4	/	4/*m* or 422	/	×	/	×
408	129 (*P*4/*n*2_1_/*mm*)	4/*mmm*	4*mm*	4/*mmm*	4*mm* or 4/*mmm*	×	×	×	×
*Equivalent Hall numbers:*		409 (*P*4/*n*2_1_/*mm*) → 408							
410	130 (*P*4/*n*2_1_/*cc*)	4/*mmm*	4	/	4/*m* or 422	/	×	/	×
*Equivalent Hall numbers:*		411 (*P*4/*n*2_1_/cc) → 410							
412	131 (*P*4_2_/*m*2/*m*2/*c*)	4/*mmm*	*mm*2	/	4̅2*m* or *mmm*	/	×	/	×
413	132 (*P*4_2_/*m*2/*c*2/*m*)	4/*mmm*	*mm*2	/	4̅2*m* or *mmm*	/	×	/	×
414	133 (*P*4_2_/*n*2/*b*2/*c*)	4/*mmm*	*mm*2	/	4̅2*m* or *mmm*	/	×	/	×
*Equivalent Hall numbers:*		415 (*P*4_2_/*n*2/*b*2/*c*) → 414							
416	134 (*P*4_2_/*n*2/*n*2/*m*)	4/*mmm*	*mm*2	/	4̅2*m* or *mmm*	/	×	/	×
*Equivalent Hall numbers:*		417 (*P*4_2_/*n*2/*n*2/*m*) → 416							
418	135 (*P*4_2_/*m*2_1_/*b*2/*c*)	4/*mmm*	*mm*2	/	4̅2*m* or *mmm*	/	×	/	×
419	136 (*P*4_2_/*m*2_1_/*n*2/*m*)	4/*mmm*	*mm*2	/	4̅2*m* or *mmm*	/	×	/	×
420	137 (*P*4_2_/*n*2_1_/*m*2/*c*)	4/*mmm*	*mm*2	/	4̅2*m* or *mmm*	/	×	/	×
*Equivalent Hall numbers:*		421 (*P*4_2_/*n*2_1_/*m*2/*c*) → 420							
422	138 (*P*4_2_/*n*2_1_/*c*2/*m*)	4/*mmm*	*mm*2	/	42̅*m* or *mmm*	/	×	/	×
*Equivalent Hall numbers:*		423 (*P*4_2_/*n*2_1_/*c*2/*m*) → 422							
424	139 (*I*4/*m*2/*m*2/*m*)	4/*mmm*	4*mm*	/	4/*mmm*	/	4*mm* or 4/*mmm*	/	×
425	140 (*I*4/*m*2/*c*2/*m*)	4/*mmm*	4*mm*	/	4/*mmm*	/	4*mm* or 4/*mmm*	/	×
426	141 (*I*4_1_/*a*2/*m*2/*d*)	4/*mmm*	*mm*2	/	/	/	4̅2*m* or *mmm*	/	/
*Equivalent Hall numbers:*		427 (*I*4_1_/*a*2/*m*2/*d*) → 426							
428	142 (*I*4_1_/*a*2/*c*2/*d*)	4/*mmm*	*mm*2	/	/	/	4̅2*m* or *mmm*	/	/
*Equivalent Hall numbers:*		429 (*I*4_1_/*a*2/*c*2/*d*) → 428							

	**Trigonal**								
430	143 (*P*3)	3	3	3	3	3	3	3	3
431	144 (*P*3_1_)	3	1	/	/	1	/	/	1
432	145 (*P*3_2_)	3	1	/	/	1	/	/	1
433	146 (*R*3)	3	3	/	/	3	/	/	3
435	147 (*P*3̅)	3̅	3	3̅	3 or 3̅	×	×	×	×
436	148 (*R*3̅)	3̅	3	/	/	3̅	/	/	3 or 3̅
438	149 (*P*312)	32	3	32	3 or 32	×	×	×	×
439	150 (*P*321)	32	3	32	3 or 32	×	×	×	×
440	151 (*P*3_1_12)	32	1	/	/	2	/	/	1 or 2
441	152 (*P*3_1_21)	32	1	/	/	2	/	/	1 or 2
442	153 (*P*3_2_12)	32	1	/	/	2	/	/	1 or 2
443	154 (*P*3_2_21)	32	1	/	/	2	/	/	1 or 2
444	155 (*R*32)	32	3	/	/	32	/	/	3 or 32
446	156 (*P*3*m*1)	3*m*	3*m*	3*m*	3*m*	3*m*	3*m*	3*m*	3*m*
447	157 (*P*31*m*)	3**m**	3*m*	3*m*	3*m*	3*m*	3*m*	3*m*	3*m*
448	158 (*P*3*c*1)	3*m*	3	/	3	/	3	/	3
449	159 (*P*31*c*)	3*m*	3	/	3	/	3	/	3
450	160 (*R*3*m*)	3*m*	3*m*	/	/	3*m*	/	/	3*m*
452	161 (*R*3*c*)	3*m*	3	/	/	/	/	/	3
454	162 (*P*3̅12/*m*)	3̅*m*	3*m*	3̅*m*	3*m* or 3̅*m*	×	×	×	×
455	163 (*P*3̅12/*c*)	3̅*m*	3	/	32 or 3̅	/	×	/	×
456	164 (*P*3̅2/*m*1)	3̅*m*	3*m*	3̅*m*	3*m* or 3̅*m*	×	×	×	×
457	165 (*P*3̅2/*c*1)	3̅*m*	3	/	32 or 3̅	/	×	/	×
458	166 (*R*3̅2/*m*)	3̅*m*	3*m*	/	/	3̅*m*	/	/	3*m* or 3̅*m*
460	167 (*R*3̅2/*c*)	3̅*m*	3	/	/	/	/	/	32 or 3̅

	**Hexagonal**								
462	168 (*P*6)	6	6	6	6	6	6	6	6
463	169 (*P*6_1_)	6	1	/	/	/	/	/	1
464	170 (*P*6_5_)	6	1	/	/	/	/	/	1
465	171 (*P*6_2_)	6	2	/	/	2	/	/	2
466	172 (*P*6_4_)	6	2	/	/	2	/	/	2
467	173 (*P*6_3_)	6	3	/	3	/	3	/	3
468	174 (*P*6̅)	6̅	3	6̅	3 or 6̅	×	×	×	×
469	175 (*P*6/*m*)	6/*m*	6	6/*m*	6 or 6/*m*	×	×	×	×
470	176 (*P*6_3_/*m*)	6/*m*	3	/	3̅ or 6̅	/	×	/	×
471	177 (*P*622)	622	6	622	6 or 622	×	×	×	×
472	178 (*P*6_1_22)	622	1	/	/	/	/	/	2
473	179 (*P*6_5_22)	622	1	/	/	/	/	/	2
474	180 (*P*6_2_22)	622	2	/	/	222	/	/	2 or 222
475	181 (*P*6_4_22)	622	2	/	/	222	/	/	2 or 222
476	182 (*P*6_3_22)	622	3	/	32	/	3 or 32	/	×
477	183 (*P*6*mm*)	6*mm*	6*mm*	6*mm*	6*mm*	6*mm*	6*mm*	6*mm*	6*mm*
478	184 (*P*6*cc*)	6*mm*	6	/	6	/	6	/	6
479	185 (*P*6_3_*cm*)	6*mm*	3*m*	/	3*m*	/	3*m*	/	3*m*
480	186 (*P*6_3_*mc*)	6*mm*	3*m*	/	3*m*	/	3*m*	/	3*m*
481	187 (*P*6̅*m*2)	6̅*m*2	3*m*	6̅*m*2	3*m* or 6̅*m*2	×	×	×	×
482	188 (*P*6̅*c*2)	6̅*m*2	3	/	32 or 6̅	/	×	/	×
483	189 (*P*6̅2*m*)	6̅*m*2	3*m*	6̅*m*2	3*m* or 6̅*m*2	×	×	×	×
484	190 (*P*6̅2*c*)	6̅*m*2	3	/	32 or 6̅	/	×	/	×
485	191 (*P*6/*m*2/*m*2/*m*)	6/*mmm*	6*mm*	6/*mmm*	6*mm* or 6/*mmm*	×	×	×	×
486	192 (*P*6/*m*2/*c*2/*c*)	6/*mmm*	6	/	6/*m* or 622	/	×	/	×
487	193 (*P*6_3_/*m*2/*c*2/*m*)	6/*mmm*	3*m*	/	3̅*m* or 6̅*m*2	/	×	/	×
488	194 (*P*6_3_/*m*2/*m*2/*c*)	6/*mmm*	3*m*	/	3̅*m* or 6̅*m*2	/	×	/	×

a Results for all settings compatible with a layered structure (*e.g.*, discarding cubic space groups) and for different *n*_c_ in the B-LM conventional cell, for systems of Category I. See sections 5.2and 5.3 to apply the results of this table to Categories II and III. For each space group all inequivalent settings are considered and labeled by their Hall number. The bulk (*G*_b_) and layer-invariant (*G*_I_) point groups are also provided. / and × indicate that a LM with given Hall setting and *n*_c_ cannot exist with our assumptions of being an MDO polytype (see sections 5.1and 5.2 for more details). Rhombohedral structures are only considered in their hexagonal setting.

For an ML-LM with *N* > 2, although the optical activity (discussed in Section 2) and the degeneracies depend only on the point group, in general, the previous considerations on when we can define pure C and LB modes cannot be directly applied.

Not only does *G*_*N*_ often differ from *G*_b_ (*e.g.*, in MoS_2_, hBN), it might also belong to another crystal system, and the degeneracies of the modes might be different in B-LM and ML-LM. *E.g.*, B-WTe_2_ is orthorhombic (space group *Pmn*2_1_, Hall number 155), but for all *N* the ML-WTe_2_ point group is always m, a monoclinic point group. In other cases, this occurs only for some *N*, like for ZnCl_2_ (tetragonal bulk, Hall number 420), where the ML-LM point group is 4̅2*m* (tetragonal) for odd *N*, but is *mmm* (orthorhombic) for even *N*.

**Figure 3:**
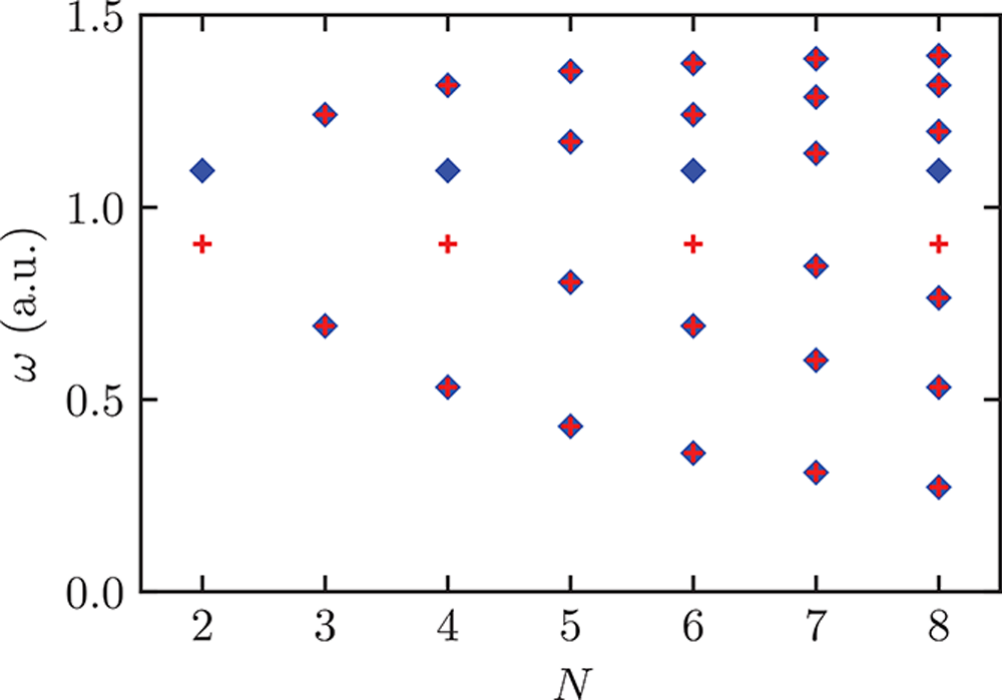
C-modes fan diagram for ZnCl_2_ obtained by solving eq 1. The phonon frequency is normalized to the mean of the two frequencies for *N* = 2. Red plus signs and blue diamonds denote C modes along the *x* and *y* in-plane directions, respectively. Both B-ZnCl_2_ and ML-ZnCl_2_ with odd *N* have tetragonal symmetry, and the two C modes are always degenerate. However, ML-ZnCl_2_ with even *N* have a reduced orthorhombic symmetry, which removes the degeneracy between some of the C modes.

The C modes are degenerate whenever the ML has an *n*-fold rotation axis with *n* > 2.^59,60^ Thus, the degeneracies vary with *N* in the case of ZnCl_2_, as illustrated in Figure 3. For even *N* there is a two-fold rotation axis and the C modes are non-degenerate, while a four-fold one exists for odd *N*, so that C modes become degenerate. This behavior can be used as an additional fingerprint of the material. More generally, the C modes degeneracy can be obtained for any LM from Table 3 by looking at the ML point groups and checking if they include a *n*-fold axis with *n* > 2.

### 1.4 Optical Activity of a Multilayer

Once the point group of an ML-LM and its normal modes (frequencies and eigenvectors) are known, one can assess its Raman or IR activity by projecting the normal modes onto the different irreducible representations of the point group (listed in standard crystallography references^78−80^) to understand to which one they belong. In particular, apart from accidental degeneracies, a normal mode belongs to only one irreducible representation,^81^ provided that pairs of complex representations that are conjugates of each other are grouped together because of time-reversal symmetry. Thus, the following expectation value will be 1 for the irreducible representation γ, with characters χ^(γ)^(*g*), to which the normal mode (ν,*n*) belongs, and 0 for all others:^81^
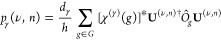
4where **U**^(ν,*n*)^ is a vector collecting the displacements **u**^(ν,*n*)^(*l*) of the layers obtained by solving eq 2, *d*_γ_ is the dimension of the representation, *h* the order of the point group, and *Ô*_*g*_ the operator associated with the symmetry element *g* (all these are tabulated for all point groups). From the knowledge of the representation γ for which *p*_γ_(ν,*n*) = 1, we can determine if the mode is Raman- and/or IR-active depending on whether the representation transforms as the components of a vector (*x*, *y*, *z*) or of a quadratic form (*x*^2^, *y*^2^, *xz*, ...), respectively. Additionally, if there exists at least one quadratic form associated with γ that does not involve the *z* coordinate, the mode should also be visible in a back-scattering Raman geometry, as the light polarization vector in a back-scattering experiment with light propagating along *z* cannot have a *z* component.

To showcase the application of the method, Tables 4 and 5 report the results obtained for all point groups when a single force-constant tensor is sufficient, so that the analytical expressions of the previous section can be adopted. In particular, for each mode of a *N*-layer ML-LM with point group *G*_*N*_, we indicate the irreducible representation to which it belongs, together with its IR/Raman activity, and whether the mode is visible in a Raman spectroscopy experiment with a back-scattering geometry. The overall number of IR/Raman-active modes is in agreement with general predictions for rigid-layer vibrations of ML-LM.^59,60^ We note that, in addition to the results of refs (59 and 60), we also derived here the ML point group starting from the B-LM symmetry properties. A full analysis for any input LM including when more than one force-constant tensor is needed is performed by our online tool.

**Table 4:**
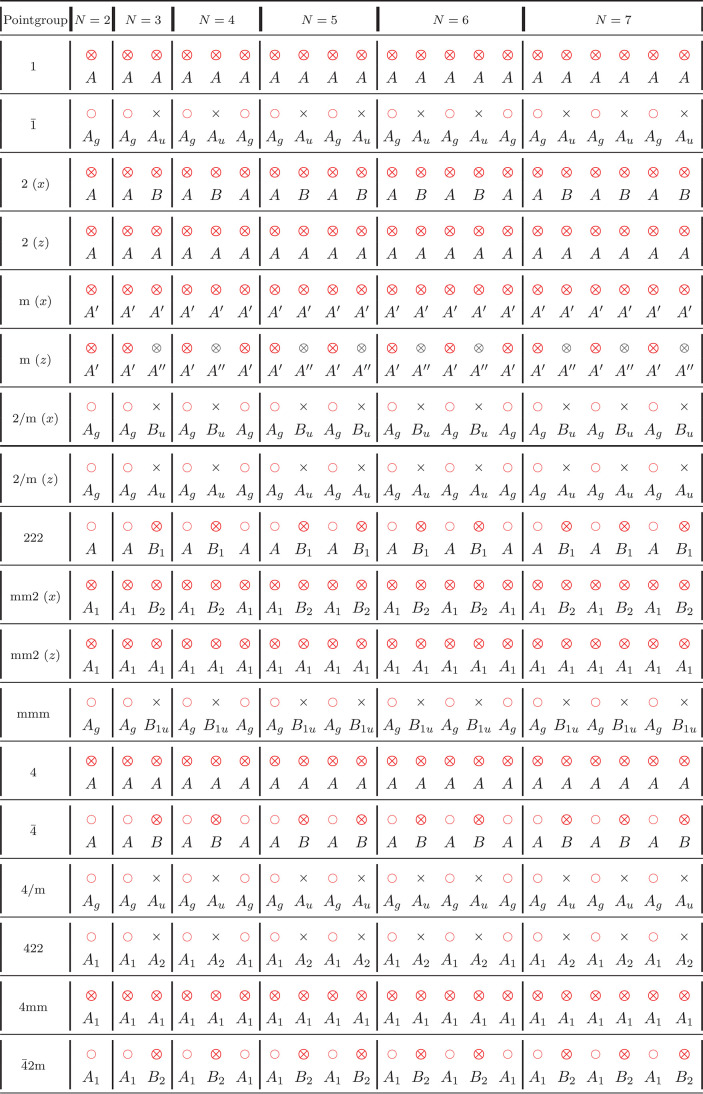

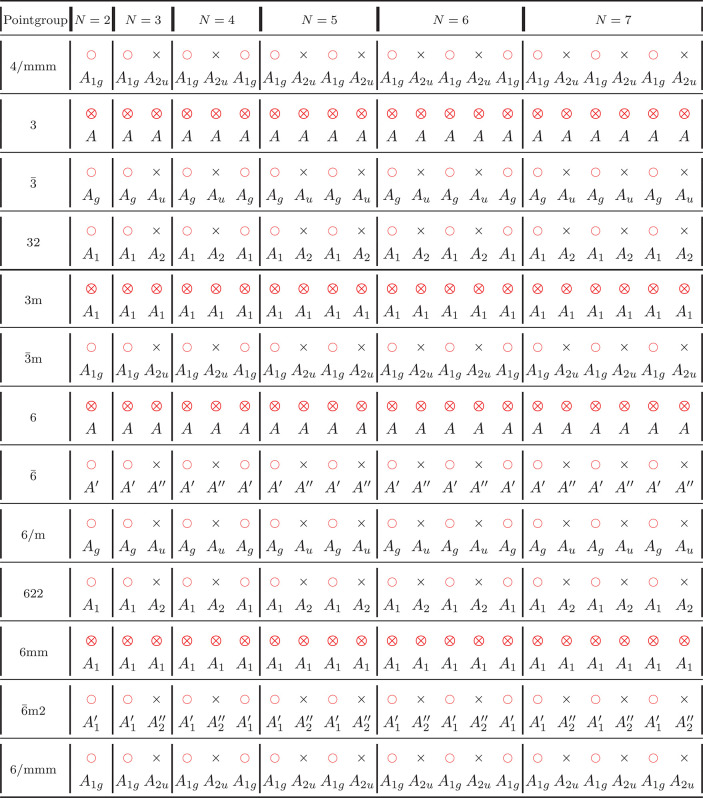
LBM Classification for NL-MLs According to Their Point Group *G*_*N*_^a^

a For a given point group and *N*, the modes are reported from left to right in order of increasing frequency. Raman-active modes are denoted as ○, infrared (IR)-active modes as ×, and those that are both Raman- and IR-active are denoted as ⊗. A red symbol indicates that the mode can be detected in a back-scattering Raman experiment orthogonal to the layers. The irreducible representation to which each mode belongs is also reported. Whenever necessary, different orientations of the principal symmetry element with respect to the layering direction (*z*) are considered and specified in parentheses. In cases where it is not possible to decouple C and LB modes, we still report them, and we note that the mode assignment coincides with that of the corresponding mixed mode in Table 5.

**Table 5:**
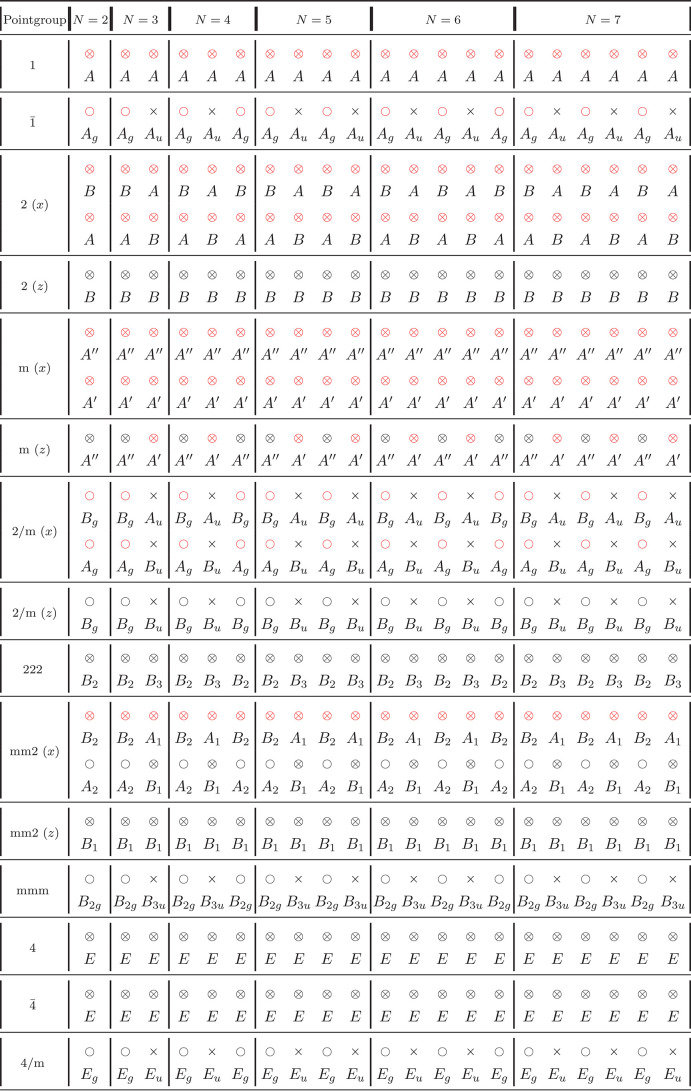

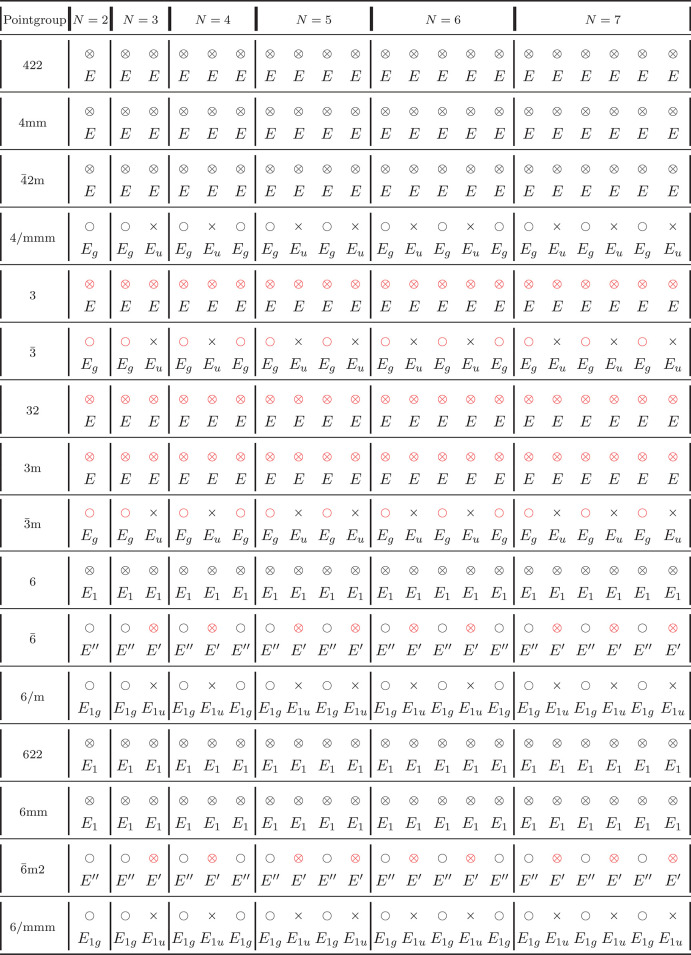
C Modes Classification for NL-MLs According to Their Point Group *G*_*N*_^a^

a For a given point group and *N*, the modes are reported from left to right in order of increasing frequency. Raman-active modes are denoted as ○, infrared (IR)-active modes as ×, and those that are both Raman- and IR-active are denoted as ⊗. A red symbol indicates that the Raman mode can be detected in a back-scattering Raman experiment orthogonal to the layers. The irreducible representation to which each mode belongs is also reported. Whenever necessary, different orientations of the principal symmetry element with respect to the layering direction (*z*) are considered and specified in parentheses. In cases where it is not possible to decouple C and LB modes, we still report them, and we note that the mode assignment coincides with that of the corresponding mixed mode in Table 4. Only modes along the first principal direction are reported if the pattern of Raman/IR activity is the same as for modes along second direction, and the irreducible representations differ just by a naming convention (*e.g.*, *B*_2_*versusB*_3_). Otherwise, displacements in both principal directions are shown.

## 2 Results

We now show with a few examples how to use this approach to reconstruct the fan diagram and the pattern of modes detectable in IR or Raman spectroscopy.

Let us start with the case of MoS_2_ and black phosphorus (BP). As previously discussed, the ML point group is 6̅*m*2 for odd *N* and 3̅*m* for even *N*.

**Figure 4:**
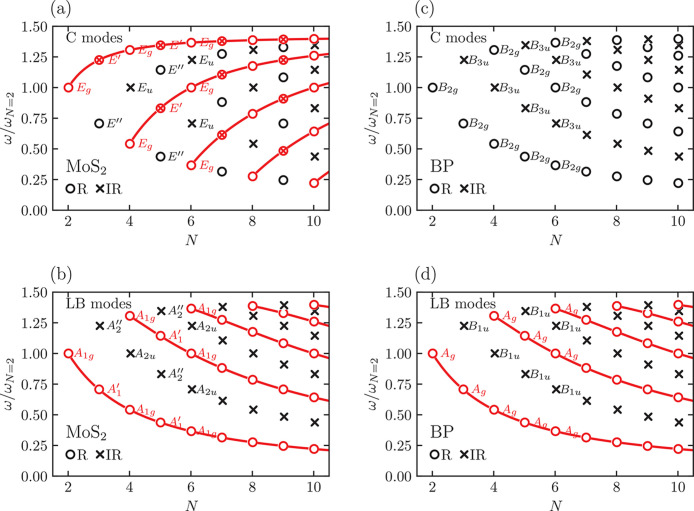
Fan diagram for C modes (panels a, c) and LBMs (panels b, d) for ML-MoS_2_ (panels a, b) and ML-BP (panels c, d). Open circles indicate Raman-active modes and crosses indicate IR-active ones. Red symbols denote Raman-active modes that are detectable in back-scattering geometry. For each mode with *N* ≤ 6, the corresponding irreducible representation of the point group is shown (in the case of ML-BP two non-degenerate sets of C modes exits, but only one of them is reported with the corresponding irreducible-representation names). Red lines are guides to the eye, following the pattern of Raman-active modes visible in back-scattering. The frequency ω on the *y* axis is normalized to the frequency ω_*N*=2_ of the corresponding mode in 2L, that is different between C and LB modes.

Figure 4a,b plots the fan diagrams for the C and LB modes of ML-MoS_2_ as a function of *N*, where the assignment of the modes is obtained by considering the appropriate entries in Tables 4 and 5. These reproduce the experiments in refs (31 and 35).

We then consider ML-BP, whose bulk space group is *A*2_1_/*b*2/*m*2/*a* (space group 64, Hall number 306 for the shortest in-plane vector along the second axis), *n*_c_ = 2, and the corresponding ML-BP point group is *mmm*, both for even and odd *N*. Figure 4c,d reports the corresponding fan diagrams, reproducing the experiments of ref (48). We note that, in this case, C modes are not visible in back-scattering, consistent with ref (48).

**Figure 5:**
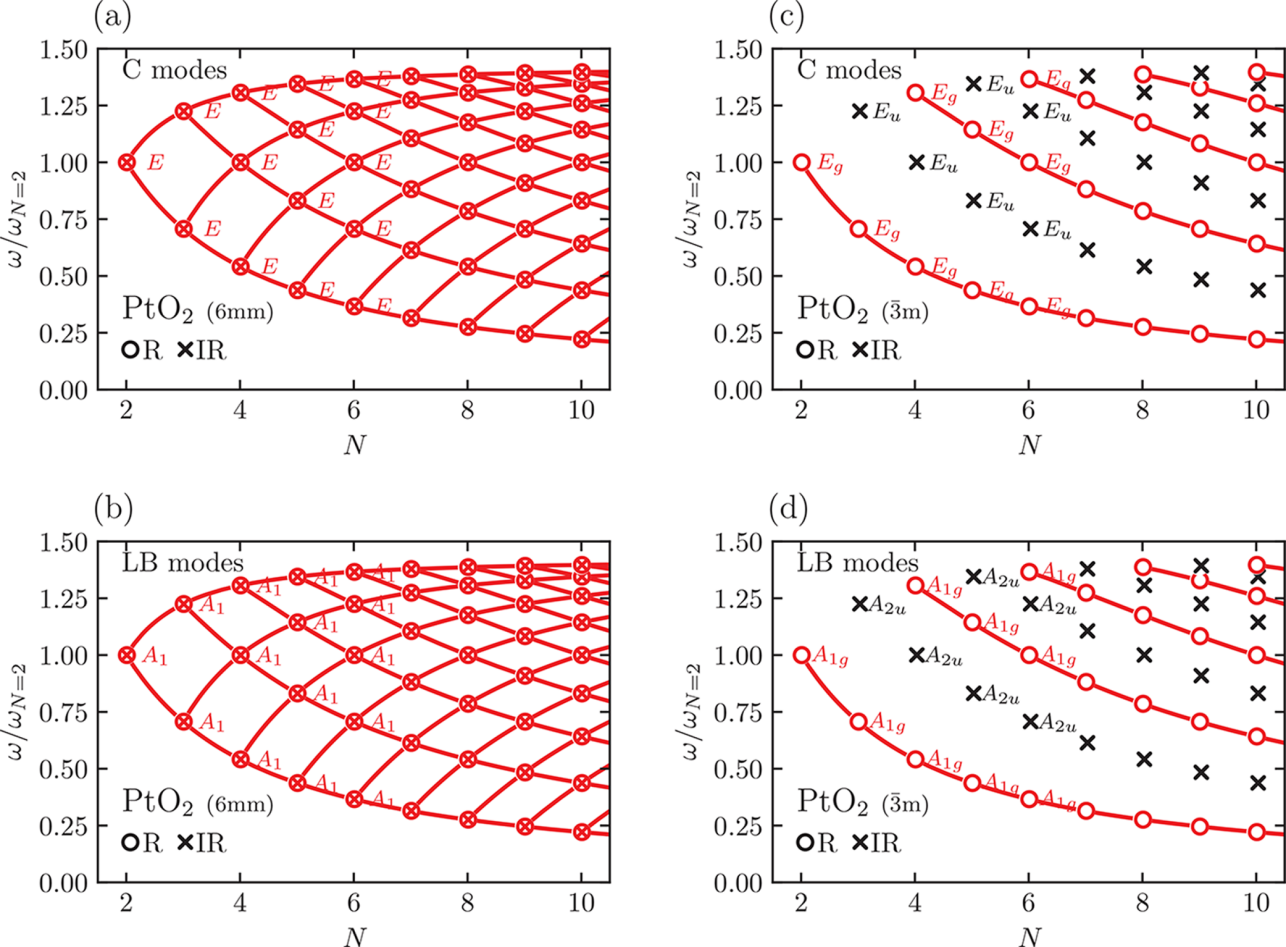
Fan diagram for C modes (panels a, c) and LBMs (panels b, d) of ML-PtO_2_ for two different bulk allotropes with space group *P*6_3_*mc* (panels a, b, point group 6*mm*) and *P*3̅*m*1 (panels c, d, point group 3̅*m*). See Figure 4 for the meaning of symbols and colors.

As a further example, Figure 5 shows the fan diagram of PtO_2_, which can crystallize in at least two different allotropes that differ only in their layer-stacking sequences.^82^ One phase has space group *P*6_3_*mc* (space group 186, Hall number 480) with *n*_c_ = 2; the other has *n*_c_ = 1 and space group *P*3̅*m*1 (space group 164, Hall number 456). In the first case, the ML-PtO_2_ point group is always 3*m* as reported in Table 3, so that all C and LB modes are Raman-active in back-scattering (see Tables 4 and 5). In the second case, the point group is 3̅*m* for every *N*, and the pattern of Raman-active modes is in Figure 5c,d. Because the pattern is different from the first phase, this implies that the pattern of Raman-active modes detectable in back-scattering can be used as a fingerprint to recognize the stacking sequence and symmetry properties of a given ML-PtO_2_.

## 3 Online Tool

**Figure 6:**
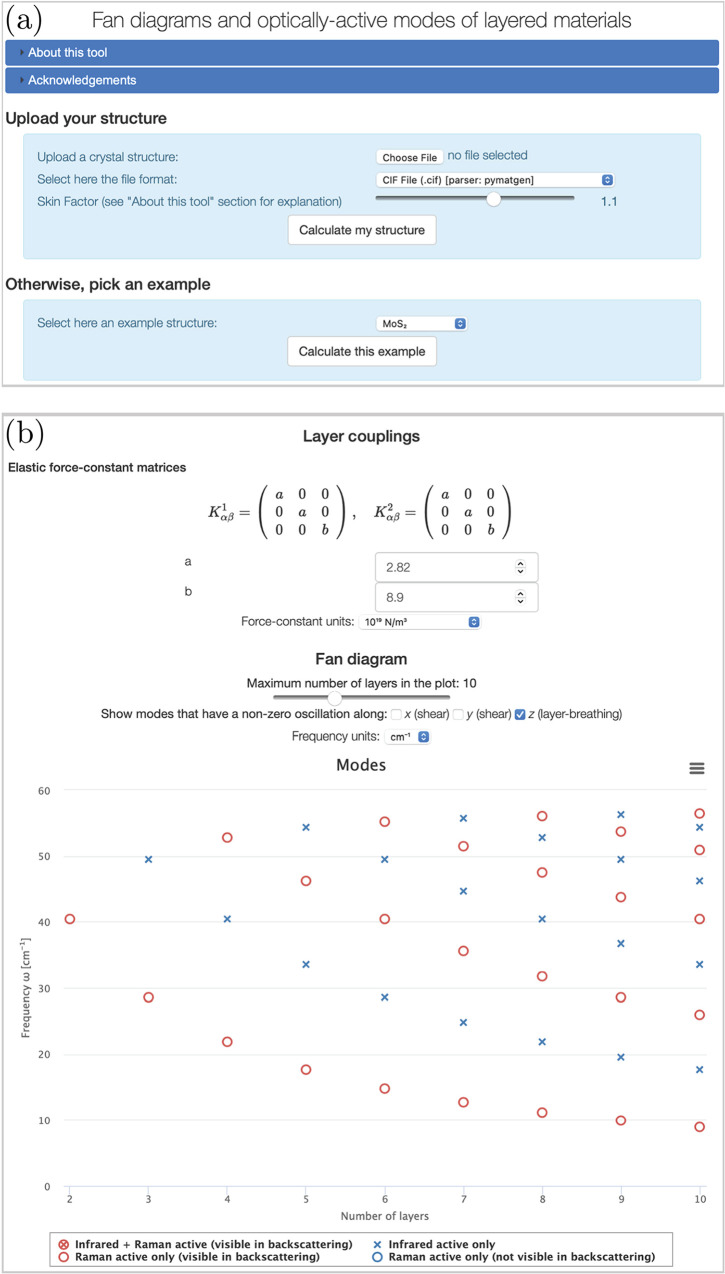
Screenshots of the online tool implementing the algorithms of this paper, available on the Materials Cloud^55^ Work/Tools section. (a) Selection page, where it is possible to upload a structure in a number of common formats, or to select an example. (b) Part of the output page with the resulting fan diagram for a material, in this case MoS_2_, where the option to show only LBMs has been selected. The output page of the tool can display much more information, like visualizations of the crystal structure of B-LM and of the layers, the coincidence operation of the ML-LM, and the symmetry analysis for the B-LM, 1L-LM, and ML-LM for all possible *N*.

In order to make the aforementioned algorithm readily available, we implemented it in an online web tool, published on the Materials Cloud web platform^55^ at https://materialscloud.org/work/tools/layer-raman-ir. This does not require any installation and works directly in the browser. In the first selection page, shown in Figure 6a, the user can upload the bulk crystal structure of a LM in a number of common formats, leveraging the parsers implemented in the ASE^83^ and pymatgen^84^ libraries. A “skin factor” parameter *f* can also be selected to tune the bond-detection algorithm. In particular, the tool considers two atoms A and B bonded if their distance is <*f*(*r*_A_ + *r*_B_), where *r*_A_ and *r*_B_ are the corresponding covalent atomic radii from ref (85). Alternatively, it is possible to choose among a few selected examples that we provide as demonstrations.

Once the bulk structure is selected or uploaded, the tool performs computations in the background and produces an output page. It first computes the bonds and then detects the disconnected lower-dimensional components. Once these are determined, the tool checks that all these components are two-dimensional and identical between them (using the pymatgen code^84^ and, in particular, the structure_matcher module, to compare layers, check if they are identical within a numerical threshold, and determine which coincidence operation brings one onto the other). It then rotates the whole structure so that the stacking axis is along *z* and computes the coincidence operation between each pair of layers in the conventional cell, verifies that the system satisfies the hypotheses of this paper (same coincidence operation between any pair of consecutive layers) and assigns one of the three categories described in Figure 1. If any of the steps does not succeed, the tool displays a message informing that the structure does not satisfy the assumptions. After this geometry analysis, the tool determines the symmetry of B-LM and 2L-LM, thus, the number and shape of the force-constant matrices. Extending the assumptions used here to produce Tables 4 and 5, the tool also works in the case in which the force constant *K* and the rotational part (proper or improper) of the coincidence operation *R* do not commute, such as, *e.g.*, in WTe_2_ and ZnCl_2_, where force-constant matrices between successive layer pairs are related by symmetry, but are not identical. The output page then includes relevant information on the structure (interactive visualizations of B-LM and 1L-LM, information on coincidence operation), and shows the independent components of the force-constant matrices. An initial random value for these components is provided, chosen to be in the range of those typically occurring in LMs, but these can be changed interactively (*e.g.*, to fit experimental data, or to use values obtained from first-principles). The tool then computes the corresponding fan diagram, including the optical activity for IR and Raman spectroscopy. Multiple units are supported both for the force constants and for the phonon frequencies. A screenshot of the resulting fan diagram as provided by the tool (including the section to select the force-constant parameters) is in Figure 6b.

## 4 Conclusions

We presented an approach to predict the spectroscopic fingerprints of layered materials composed of repetitions of the same layer. We explained how to obtain, using symmetry considerations, the point group of a finite ML, knowing the space group and the Hall setting of the bulk, and provided a table for all possible space groups and settings. We derived the vibrational modes for any number of layers using a tensorial linear chain model. We then exploited these results to associate each normal mode to a given irreducible representation of the point group of the ML, to assess the corresponding optical activity and, thus, to obtain the fan diagram and the pattern of modes that are detectable in IR and Raman spectroscopy. We demonstrated with various examples that this approach can distinguish different stacking sequences of a given LM, and provides stringent conditions on the symmetry properties of MLs.

We also provided an easy-to-use online web tool that enables users to upload a bulk LM of their choice (accepting a variety of common crystal-structure formats) and to perform all operations to obtain and to display interactively the corresponding fan diagram, even beyond some of the approximations used in this paper (like those used in Tables 4 and 5). The tool is available on the Materials Cloud web platform^55^ at https://materialscloud.org/work/tools/layer-raman-ir and it is fully open-source (the code is at https://github.com/epfl-theos/tool-layer-raman-ir). This will guide computational and experimental researchers interested in studying or interpreting fan diagrams of LMs.

## 5 Methods

### 5.1 Compatibility Relations of Fractional Translations

We consider a space group operation defined by the following expression for the coordinate transformation:
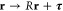
5where *R* is an orthogonal matrix. The translation **τ** is applied by convention after the application of the *R* matrix. We refer to *R* as the rotation part of the transformation (either proper or improper rotation, *e.g.*, a mirror operation). A non-zero **τ** is called a fractional translation.

Because we focus on LMs stacked along the *z* axis, we consider only the τ_*z*_ component. In order for an operation with a non-zero τ to be compatible with a LM with *n*_c_ layers in the conventional cell, the product *n*_c_·τ_*z*_ must be an integer: *e.g.*, if we consider a space group with a 3_1_ screw axis along *z*, it might be possible to construct a LM with this space group having 3, 6, ... layers in the B-LM conventional cell, but it is not possible to define an ML system having *n*_c_ = 1, 2, 4, 5, .... In Table 3 we indicate therefore with a slash (/) any space group that contains at least one incompatible operation for a given *n*_c_.

Non-vanishing τ (in the case of MDO polytypes) are therefore admissible only when *n*_c_ = 2, 3, 4, 6. This limit follows from the usual crystallographic conditions for which, *e.g.*, if we rotate a layer by an arbitrary angle, the next one cannot be periodic with the same unit cell, except for a few angles (see Chapters 1 and 2 of ref (70)).

### 5.2 Grouping Fractional Translations of Layer-Order-Changing Operations: Category I

As discussed in the main text, in order to obtain *G*_*N*_ of an ML-LM we need to identify the B-LM LOC operations compatible with it. These, together with the elements of the layer-invariant point group *G*_I_, will form the *G*_*N*_ that we seek.

We now consider independently the 3 categories of Figure 1. In Category II, there are no LOC operations, therefore *G*_*N*_ = *G*_I_. We focus in the rest of this section on Category I and we show in Section 5.3 that for Category III we can adapt the results of Category I.

We consider the subset of LOC operations of a given space group (and setting), defined as those that swap the orientation of the *z* axis, *i.e.*, where the third column of the rotation matrix *R* is the vector (0, 0, −1) . Focusing only on the third coordinate *z* of a coordinate vector **r** and using eq 5, the transformation will therefore read:
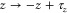
6Let us first fix the origin of our coordinate system by setting it on the inversion plane of the *i*th LOC operation, which will then have no fractional translation along the vertical direction (τ_*z*_^*i*^ = 0).

If we now choose another LOC operation, say the *j*th, we might need to associate with it a non-zero τ_*z*_^*j*^. In order to connect the coordinate of the inversion planes *z̃*_*j*_ for this *j*th LOC to its τ_*z*^*j*^_, we note that the *j*th transformation can be equivalently interpreted as the combination of the following operations: (1) translating one inversion plane at *z* = *z̃*_*j*_ to *z* = 0 with a transformation *z* → *z* – *z̃*_*j*_; (2) applying the inversion transformation about the plane that is now at *z* = 0, therefore changing the sign of the *z* coordinate, so that the combined transformation reads *z* → −(*z* – *z̃*_*j*_); and (3) shifting back the inversion plane to its original position by adding *z̃*_*j*_ to the third coordinate. The total transformation is thus *z* → −(*z* – *z̃*_*j*_) + *z̃*_*j*_ = −*z* + 2*z̃*_*j*_. Comparing this with eq 6, we obtain τ_*z*_^*j*^ = 2*z̃*_*j*_.

As we discussed earlier (see Figure 1), for Category I inversion centers can only be on a layer or on a middle plane. Having also chosen earlier the origin on one of these planes, the *z̃*_*j*_ coordinate of any center (in fractional coordinates) can, thus, only be at position *z̃*_*j*_ = *k*/2*n*_c_, with . In the case of two layers A and B in the conventional cell (*n*_c_ = 2), centers will be at *z̃*_*j*_ = 0 (on layer A), *z̃*_*j*_ = 1/4 (between layer A and layer B), *z̃*_*j*_ = 1/2 (on layer B) or *z̃*_*j*_ = 3/4 (between layer B and layer A in the next unit cell). Thus, fractional translations for any inversion plane can only assume values τ_*z*_ = *k*/*n*_c_, with .

We can then use the information on τ_*z*_^*j*^ to group all LOC operations in sets that share the same inversion plane(s), distinguishing those operations having τ_*z*_ = 2*h*/*n*_c_ (with ), and, thus, inversion on a layer plane, from those having τ_*z*_ = (2*h*+1) /*n*_c_, with inversion on a middle plane. If *n*_c_ is odd, the two sets are equivalent (*i.e.*, each LOC transformation with inversion on a layer plane can be also written as an operation with inversion on a middle plane and a different τ). *E.g.*, in the case *n*_c_ = 3, one of the two groups is {τ_*z*_ = 0, 2/3, 4/3, 6/3 = 2, ...} and the second {τ_*z*_ = 1/3, 3/3 = 1, 5/3, 7/3, ...}. Remembering that adding an integer to τ_*z*_ does not change the operation, we have that 4/3 is equivalent to 1/3, 2 to 0, 5/3 to 2/3, and so on, so that both sets coincide with {0, 1/3, 2/3}. If *n*_c_ is even, instead, there are two separate sets of τ_*z*_, giving rise to transformations having inversion either on layer planes or middle planes. *E.g.*, for *n* = 4, one such set contains {τ_*z*_ = 0, 1/2} and the other {τ_*z*_ = 1/4, 3/4}.

For each τ_*z*_ in one of these sets we can construct a potential point group *G*_*N*_^τ_*z*_^ by adding to *G*_I_ all LOC operations with fractional translation τ_*z*_. In order to be consistent with our initial assumption of a layered structure with *n*_c_ identical layers per cell and with the same relation between nearest layers (MDO polytypes), all possible *G*_*N*_^τ_*z*_^ should be identical for all τ_*z*_ belonging to the same set. This stems from the fact that for Category I MDO polytypes all layer and middle planes are equivalent. If this is not the case, we indicate it with a cross (×) in Table 3. *E.g.*, in the case of space group *P*1̅ (Hall number 2) the B-LM point group is 1̅, whereas *G*_I_ is 1. For *n*_c_ = 3, considering LOC operations with τ_*z*_ = 0 would add the 1̅ operation and give *G*_*N*_^0^ = 1̅. However, considering operations with τ_*z*_ = 1/3 (or τ_*z*_ = 2/3) would give rise to a different *G*_*N*_^1/3^ = 1 (*G*_*N*_^2/3^ = 1), because *P*1̅ has no LOC operations with these τ_*z*_, thus *G*_*N*_^τ^_*z*_ = *G*_I_ in this case. These point groups (1 and 1̅) are not the same. Therefore, we mark this with ×, indicating that it is not possible to construct an ML with symmetry *P*1̅ and *n*_c_ = 3 identical layers with the same relation between each pair.

We summarize the results as follows: if *n*_c_ is odd, we can either obtain a / or a ×, or there will be only one possible value for *G*_*N*_, independent of *N*. When *n*_c_ is even, the only difference is that, in general, there can be two possible choices for *G*_*N*_. Which value is taken in the finite ML depends on the parity of *N*: the only LOCs compatible with a finite ML are those with symmetry plane at its center (a middle plane for even *N* or a layer plane for odd *N*). Therefore, in these cases, the two possible point groups alternate as a function of *N*.

In the example of Figure 2, the Hall number is 242 (Hall symbol *P*2/*c*2/*m*2_1_/*m*), *n*_c_ = 2, and *G*_I_ = *m*. Because we have a 2_1_ vertical axis, *n*_c_ must be even, and in Table 3 there is a / for all odd *n*_c_. If we add LOC operations with a given fractional translation to *G*_I_, we obtain *G*_I_ = *m* for τ_*z*_ = 1/6, 1/4, 1/3, 2/3, 3/4, 5/6 (because there is no additional LOC operation with these τ_*z*_). We obtain instead 2/*m* for τ_*z*_ = 0, and *mm*2 for τ_*z*_ = 1/2. Therefore, for *n*_c_ = 2 we have two independent sets of τ_*z*_ ({0} and {1/2}), and we thus obtain the two valid options for *G*_*N*_: 2/*m* and *mm*2. However, for *n*_c_ = 4 (and similarly for larger even values of *n*_c_) we obtain a ×, because one set of τ_*z*_ {0, 1/2} (that must be equivalent for *n*_c_ = 4) would instead contain two different point groups, 2/*m* and *mm*2.

From pure symmetry considerations it is not possible to establish which of the two point groups takes place for odd or even *N*, as discussed in Figure 2, unless something is known for 1L.

### 5.3 Grouping Fractional Translations of Layer-Order-Changing Operations: Category III

If we limit ourselves to symmetry considerations (*e.g.*, for the determination of the results of Table 3), we note that Category III is equivalent to Category I. Indeed, if we consider a pair of adjacent layers in Category III, these together can be considered as a (now non-polar) “layer” of Category I. In particular, the σ–ρ plane between the pairs takes the role of the σ–ρ middle plane of Category I, and the σ–ρ plane between the layers of the pair takes the role of the λ–ρ of Category I. There are two ways of pairing adjacent layers, and changing such choice swaps the role of middle and layer planes.

Intuitively, we can understand why these two categories are equivalent with the following *Gedankenexperiment*: if the chemical bonding between the two layers in a pair becomes stronger, without changing the atomic positions (without any change to the symmetry of the system), we will eventually end up considering both layers in the pair to be chemically bonded and, therefore, part of the same rigid layer. In this case, we would have considered the system as belonging to Category I. Thus, for the purpose of knowing the possible point groups, Table 3 can still be used, with the caveat that now *n*_c_ indicates the number of pairs of layers for Category III.

We emphasize, however, that a separate treatment is needed when we consider the force constants between layers. In this case, the strength of the chemical bonds matters in determining which layers can be considered as moving rigidly, and we need to consider two different sets of force constants for the various σ–ρ planes of Category III.

In conclusion, to determine the point group of an ML with *N* layers, there are the following options:

- *N* is odd. In this case, on one of the two terminations there is only one layer in a pair. The ML loses all LOC symmetries, and *G*_*N*_ = *G*_I_.
- *N* is even. We can then map this case to Category I, considering a system with *Ñ* = *N*/2 pairs of layers as discussed above. Depending on the parity of *n*_c_ (which now indicates the number of pairs of layers in the conventional cell) we might have only one or two possibilities for the resulting *G*_*N*_. The termination of the finite ML will uniquely determine how to pair together adjacent layers.

Therefore, for Category III, there might be up to 3 different point group values as a function of *N*.

“Dimerized” systems with non-polar layers, where the interlayer distance alternates (A/B/A/B/...), are still MDO polytypes and behave like those of Category III, and the symmetry plane of the σ–ρ coincidence operation does not coincide with the layer plane. We do not consider them explicitly here (and they are quite unlikely to occur in real ML-LMs) but the online tool is able to account for these correctly, and mark them as Category III.

### 5.4 Point Group of Multilayer Layered Materials with *N* < *n*_c_

In the main text, we focused on the case *N* ≥ *n*_c_, for which we can deduce *G*_*N*_ starting from *G*_b_, and remove the operations that are not valid in an ML-LM with *N* layers. The operations that remain form *G*_*N*_. Therefore *G*_*N*_ is always a subgroup of *G*_b_.

**Figure 7:**
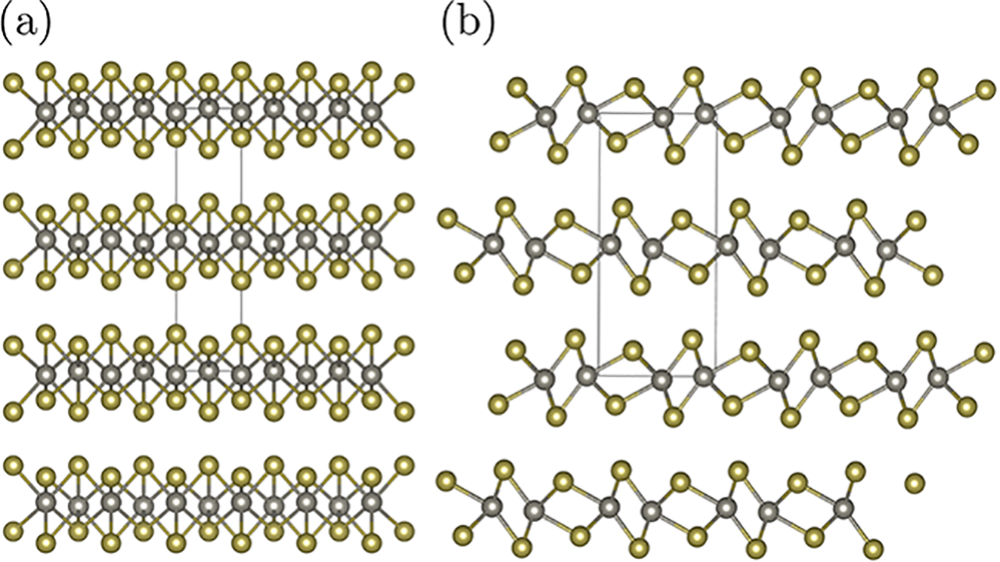
ML-WTe_2_ (COD^67^ entry ID 2310355; gray = W, yellow = Te). (a) Side view (*x*–*z* projection). (b) Side view (*y*–*z* projection). B-WTe_2_ has space group P*mn*2_1_ (number 31) with two layers in the conventional unit cell (*n*_c_ = 2; the unit cell is shown); *G*_b_ is *mm*2 (*C*_2*v*_). With the given choice of axes, the Hall setting is 155 (*Pmn*2_1_), with a mirror plane orthogonal to *x*, a glide plane orthogonal to *y*, and a 2_1_ screw axis along *z*. 1L-WTe_2_ has space group *P*2_1_/*m* (with inversion symmetry and a 2_1_ screw axis along *x*), thus *G*_*N*=1_ is 2/*m* (*C*_2*h*_). There is no group–subgroup relation between *mm*2 and 2/*m*. From Table 3, for *N* ≥ 2, the point group of any ML-WTe_2_ is *G*_*N*_ = *m* (a subgroup both of *mm*2 and of 2/*m*). Inversion symmetry, and the horizontal 2_1_ screw axis, are lost for any ML-WTe_2_ for the given stacking with a B-WTe_2_ orthorhombic cell (they might be retrieved with a different stacking having an appropriate monoclinic cell).

If *N* < *n*_c_, this group–subgroup relation is, in general, not valid anymore. When looking at the point group, *e.g.*, of a 1L, we have fewer conditions to satisfy (in particular, we remove the constraints on the specific stacking order of the layers). Therefore, in general, the 1L point group could have more operations than the ML. The examples of ML graphite and graphene are covered in the main text. As another example, we discuss WTe_2_ in the caption of Figure 7.

### 5.5 An Orthorhombic System Where Modes Are Not Purely Perpendicular or Parallel

We now consider the system of Figure 2a. 2L-LM has point group 2/*m* (monoclinic, as any ML-LM with even *N*), whereas MLs with odd *N* are orthorhombic. By inspecting the crystal structure, we deduce that the unique axis of the 2L-LM is along *y*. Therefore (see Table 2), the force-constant tensor for 2L-LM has the form:
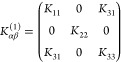
7for some non-zero values of *K*_11_, *K*_22_, *K*_33_, *K*_31_.

In addition, the coincidence operation can be written as a mirror orthogonal to *x* followed by a translation along *z*, so that if we write the coincidence operation in the form of eq 5, its (improper) rotational part *R* is:
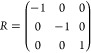
8This is not the only way to write the coincidence operation. Composing it with any bulk operation still provides a valid one. The results discussed below, however, are independent of the specific choice.

*R* and *K*^(1)^ do not commute. Therefore, force constants alternate at each interface, taking the values *K*^(1)^ and *K*^(2)^, with the latter being defined as:
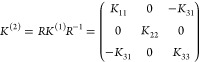
9We first observe that, if we limit ourselves to the 1×1 block along *y*, we can consider *R* and *K* as commuting. Therefore, there will be a mode with pure oscillations along *y*, *i.e.*, a pure C mode.

Let us now focus only on the *xz* subspace, and define the *xz* sub-blocks of *K*^(1)^ and *K*^(2)^ as:
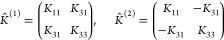
10We first note that, in 2L-LM, the *x* and *z* components mix (due to the off-diagonal *K*_31_ component), so that, as expected for a monoclinic system, we cannot define pure LB or C modes. The same happens for all even *N* (monoclinic). One might expect that for odd *N*, since the point group is instead orthorhombic, *x* (LB) and *z* (C) modes would perfectly decouple. However, this is not the case. This is verified by defining a displacement vector **U** = (*u*_*x*_(1), *u*_*z*_(1), *u*_*x*_(2), *u*_*z*_(2), ..., *u*_*x*_(*N*), *u*_*z*_(N)) ^*T*^ so that the equation of motion eq 1 can be written as −*M*ω_*n*_^2^**U** = *K̂***U**, with *K̂* having the following block form:
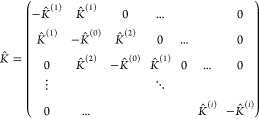
11where *K̂*^(0)^ = *K̂*^(1)^ + *K̂*^(2)^ and *i* = 1 for even *N*, while *i* = 2 for odd *N*.

Even if the *K̂*^(0)^ block is diagonal, there are still mixed *xz* components in the off-diagonal *K̂*^(1)^ and *K̂*^(2)^ blocks. Thus (independent of *N* parity) all eigenvectors have non-zero *x* and *z* components. Nevertheless, for odd *N* (orthorhombic) *K̂* commutes with the mirror operation orthogonal to *z* at the center of the ML, therefore eigenvectors can be chosen to be simultaneously eigenvectors also of this mirror operation (whereas this is not the case for even *N*). Thus, they respect the orthorhombic symmetry of MLs with odd *N*, and the optical activity is given by the irreducible representations of the corresponding orthorhombic point group.

In summary, even in the orthorhombic case we cannot define purely LB and C modes (on the *xz* subspace) and, more generally, the decoupling of the modes is determined by the crystal symmetry of 2L-LM, not of the full ML-LM. The optical activity, however, is determined by the point group of the full ML-LM, as discussed in the main text.

### 5.6 Worked-Out Example: Activity for Group 2/*m*

We now consider how to obtain the classification of the optical activity of the modes in the example of Figure 2a with *N* = 4. The symmetry of this system is described in Section 5.5 (ML-LM point group 2/*m*), and the four symmetry operations are 1 (identity), 2 (180° rotation about the *y* axis), 1̅ (inversion), and *m* (mirror plane, orthogonal to the *y* axis).

There are two force-constant tensors, *K*^(1)^ and *K*^(2)^ in eqs 7 and 9, that alternate. However, if we limit ourselves to the 1×1 subspace for the decoupled C mode with oscillations along *y*, only a single component *K*_22_ is sufficient to describe the force between any pair of layers. We can, therefore, for this specific case, use the model described earlier, limiting to α = β = 2 (*y* axis). The final equation of motion can be written in matrix form as:
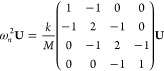
12where, as in the main text, we assume a harmonic form for *u*(*l*,*t*) = *u*(*l*) e^*i*ω_*n*_*t*^, so that *ü*(*l*,*t*) = −ω_*n*_^2^*u*(*l*,*t*); **U** is the column vector of the displacements along *y* for each layer, *i.e.*, **U** = (*u*_*y*_(*l* = 1), *u*_*y*_(*l* = 2), *u*_*y*_(*l* = 3), *u*_*y*_(*l* = 4))^*T*^; and *k* = *K*_22_.

Eq 12 is an eigenvector equation with eigenvalues ω_*n*_^2^, and can be solved to find the following 4 solutions (**U**^(*n*)^ being the corresponding eigenvectors):
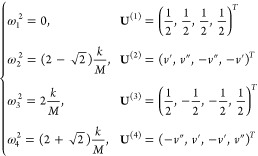
13with  and . The frequencies are the same as those obtained from eq 3.

Now that we have **U**^(*n*)^, in order to apply eq 4 we still need to get the table of the irreducible representations for the point group 2/*m*. These are found in refs (78 and 79):
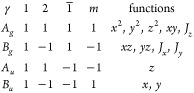
where the first row indicates the symmetry elements *g*, and the values in the table are the characters χ^(γ)^(*g*) for the 4 irreducible representations γ = *A*_*g*_, *B*_*g*_, *A*_*u*_, *B*_*u*_ of 2/*m* (they all have the same dimension *d*_γ_ = 1, and the order of the 2/*m* group is *h* = 4). *A*_*g*_ and *B*_*g*_ are Raman-active since they transform as quadratic functions, while *A*_*u*_ and *B*_*u*_ are IR-active since they transform as linear functions. Between the two Raman-active representations, only modes corresponding to *A*_*g*_ are visible in a back-scattering geometry, because there are quadratic forms (*x*^2^, *y*^2^, *xy*) that involve only the *x* and *y* coordinates.

Applying eq 4 is straightforward when we note that *Ô*_1_ is the identity; *Ô*_2_ is a 180° rotation with the axis along *y*, so it does not change the sign of displacements along *y*, but it swaps the order of the layers; *Ô*_1̅_ changes both the signs of the displacements and the order of the layers; *Ô*_*m*_ changes the signs of displacements along *y*, but not the order of the layers:
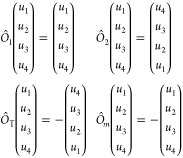
14Applying eq 4, we get that *p*_γ_(*n* = 2) and *p*_γ_(*n* = 4) are 1 only for γ = *B*_*g*_ (*i.e.*, Raman-active only), while *p*_γ_(*n* = 3) is 1 only for γ = *A*_*u*_ (*i.e.*, IR-active only). We skip *n* = 1 because this is an acoustic mode with zero frequency where all layers move by the same amount. These representations correspond to the top row of the 2/*m* (*x*) case of Table 5.

## Vocabulary

**C mode**: a mode in which layers in a multilayer oscillate as rigid units parallel to the planes
**layer-breathing mode**: a mode in which layers in a multilayer oscillate as rigid units perpendicular to the planes
**fan diagram**: a plot of the frequency of C and layer-breathing modes as a function of the number of layers in a multilayer
**coincidence operation**: a coordinate transformation that preserves distances and angles, bringing a layer of a layered material onto the next one
**crystallographic (Hall) setting**: the specification of the space group together with the choice of origin and the orientation of the symmetry elements
**maximum degree of order polytypes**: multilayer layered materials where the coincidence operation is total, *i.e.*, the same between any pair of adjacent layers
**layer-order-changing operations**: symmetry operations that change the sign of any vertical coordinate
